# Peptidoglycan mediates *Leptospira* outer membrane protein Loa22 to toll-like receptor 2 for inflammatory interaction: a novel innate immune recognition

**DOI:** 10.1038/s41598-020-79662-8

**Published:** 2021-01-13

**Authors:** Shen-Hsing Hsu, Ming-Yang Chang, Shih-Ming Lin, Yi-Ching Ko, Li-Feng Chou, Ya-Chung Tian, Cheng-Chieh Hung, Chih-Wei Yang

**Affiliations:** 1 Department of Nephrology, Kidney Research Center, Chang Gung Memorial Hospital, Chang Gung University College of Medicine, 5 Fu-Shing St., Taoyuan, 33333 Taiwan, ROC; 2 Department of Biotechnology and Bioindustry Sciences, National Cheng Kung University, Tainan, 70101 Taiwan, ROC

**Keywords:** Cytokines, Proteins, Structural biology, Diseases, Nephrology, Pathogenesis

## Abstract

Leptospirosis is an overlooked zoonotic disease caused by pathogenic *Leptospira* depended on virulence of *Leptospira* and the host–pathogen interaction. Kidney is the major organ infected by *Leptospira* which causes tubulointerstitial nephritis. *Leptospira* outer membrane contains several virulence factors and an outer membrane protein A (OmpA) like protein (Loa22) is essential for virulence. Pull-down assays suggested that Loa22 was a potential Toll-Like Receptor 2 (TLR2) binding candidates from pathogenic *Leptospira*. Confocal microscopy was employed to observe the co-localization of TLR2 and Loa22-LPGN (*Leptospira* peptidoglycan) complexes. Atomic force microscopy (AFM), side-directed mutagenesis, and enzyme-linked immunosorbent assay (ELISA) were performed to investigate the affinity between rLoa22, LPGN, and TLR2. Real time PCR was applied to measure the cytokines expression. Downstream signal transduction components were verified by western blot to evaluate the gene regulations. Mutation of two Loa22 key residues (Asp^122^ and Arg^143^) attenuated the affinities for LPGN. rLoa22-LPGN complexes were observed to co-localize with TLR2 and provoked inflammatory responses including *CXCL8/IL8*, *hCCL2/MCP-1*, and *hTNF-α*. Affinity studies suggested that Loa22-LPGN complexes elevated the affinity to TLR2 as compared to Loa22 protein. Downstream signals from TLR2 including p38, ERK, and JNK were regulated under rLoa22-LPGN complexes treatments. This study identified LPGN mediates interactions between Loa22 and TLR2 and induces downstream signals to trigger inflammatory responses. rLoa22-LPGN-TLR2 complexes reveal a novel binding mechanism for the innate immune system.

## Introduction

*Leptospira* is the pathogen of the most overlooked zoonotic diseases leptospirosis, which results in multiple-organ failure (Weil’s syndrome), especially of the kidney^1–3^. The disease is generally transmitted through contact with urine of carrier hosts in water or soil, causing infection in humans via dermal or gastrointestinal routes^4^. Several clinical symptoms include high fever, jaundice, and renal failure were observed in humans^5^. Renal proximal tubular cells are the major targets cells of *Leptospires* in the kidney^2^. Previous study showed that kidney epithelial cells pretreated with outer membrane extractions from *Leptospira* that triggered significant expression the genes that related to tubulointerstitial nephritis^6^. The functions of *Leptospira* surface-exposed antigens are likely involved in host cell adhesion and invasion^7^. *Leptospira* invades the host from the wound and multiply in the tissue, while the immune system can recognize *Leptospira* by specific surface-exposed antigens. The *Leptospira* outer membrane contains antigenic components such as lipoproteins, lipopolysaccharide (LPS)^8^, and peptidoglycans (PGN)^9^ that implicated in virulence.

In *Leptospira* research, the immunogenic outer membrane proteins of pathogenic *Leptospira* has become an important topic and among these immunogenic proteins, Loa22 was detected in pathogenic *Leptospires* but not in non-pathogenic *Leptospires*, indicating the probable involvement of this protein in virulence^10^. The domains prediction showed that Loa22 protein exhibits the signal peptide domain, N-terminal domain (residues 1–77), and an outer membrane protein A (OmpA) domain (residues 78–186; predicted as peptidoglycan-associating motif)^11^. Previous study demonstrated that Loa22 was a lipoprotein with lipidation molecules that were potent mediators of inflammatory responses^11^. Besides, the recombinant Loa22 protein was proven to interact with the extracellular matrix (ECM) including plasma fibronectin and collagen types I and IV in vitro, suggesting that the Loa22 may function as an adhesin^12^. The role of Loa22 during pathogenesis remains to be determined and the biological function and detailed mechanism involved in infection of host cells by *Leptospira* need further investigation.

The initial interactions between pathogens and host cells trigger innate immune responses at the infection site^13^. Innate immune system develops germline-encoded pattern-recognition receptors (PRRs) to sense virulence components derived from various microbes^1^. PRRs are responsible for recognizing microbe-specific molecules known as pathogen-associated molecular patterns (PAMPs)^14^. Toll-like receptors (TLRs) have been well studied in order to identify their function as PRRs^14^. TLRs are further divided into two subfamilies; cell surface (TLR1, TLR2, TLR4, TLR5, TLR6, and TLR10) and intracellular TLRs (TLR3, TLR7, TLR8, TLR9, TLR11, TLR12, and TLR13), according to their localization^13^. TLR family proteins play a pivotal role in innate immunity by recognizing conserved patterns in diverse microbial molecules^15^. Among these TLRs, TLR2 in association with TLR1 or TLR6 is essential for sensing bacterial lipoproteins and lipopeptides^16,17^. The leucine-rich repeats (LRRs) of TLRs are responsible for pattern recognition from bacterial infection and a Toll/IL-1 receptor (TIR) domain is responsible for signal transduction that inducing the inflammatory responses^13^. TLR2/1-Pam_3_CSK_4_ complex structure revealed that TLR2 associated with two fatty esters and TLR1 connected with the amide-bound lipid chain^18^. Therefore, the lipid molecules of lipoprotein were presumably in the TLR2 binding domain. In addition to the lipid domain of lipoprotein, several known structures and motifs of the TLR2-binding protein have been reported. The PorB protein from *N. meningitides* has been suggested as a TLR2 ligand and the binding mechanism was hypothesized to involve electrostatic interactions contributing to ligand/receptor interactions^19^. The BspA surface antigen from *T. forsythia* with LRR domains at N-terminus was also reported as the TLR2 ligand^20^. The pentameric B subunit of type IIb *E. coli* enterotoxin (LT-IIb-B5) uses its hydrophobic residues (Met^69^, Ala^70^, Leu^73^, and Ser^74^) to bind TLR2 directly^21^. The *Leptospira* outer membrane lipoprotein, LipL32, interacts with TLR2 through Nβ1β2 and Cα4 domains and Val^35^, Leu^36^ and Leu^263^ are involved in TLR2 interaction^22^. These TLR2 ligands use various binding mechanisms to interact with the innate immune system cell receptors inducing inflammatory responses.

It has been proven that Loa22 stimulated inflammatory responses and deletion of Loa22 from pathogenic *Leptospira* attenuated toxicity, while re-expression of the protein restores the virulence^11^. However, the pathogenic mechanisms of Loa22 are still unclear. Here, pull-down assays revealed that the TLR2 protein interacted with Loa22 from the *Leptospira* outer membrane extractions. Loa22 protein in complex with LPGN was also observed to co-localize with TLR2 on the surface of HEK293-TLR2 cell. The corresponding inflammatory responses provoked by Loa22-LPGN were measured by real-time RT-PCR and ELISA^23^. The Loa22 protein was further used to investigate interaction domains involving LPGN. In addition, we further mutated the two key OmpA domain residues, Asp^122^ and Arg^143^, and measured their relative affinities to LPGN. The interactions of Loa22 and TLR2 were analyzed using ELISA and AFM, and the role of LPGN was verified to identify the interaction between Loa22 and TLR2. It has been reported that TLR2 interacts with PGN molecules to induce inflammatory responses^24^. We hypothesized that the OmpA domain of Loa22 played vital roles in interactions with TLR2. Therefore, Loa22 was proposed to interact with LPGN molecules through the vital residues, and consequently interacts with TLR2 to induce downstream signalings and cytokines production.

## Results

### Identification of TLR2 binding candidates from pathogenic *Leptospira*

**Figure 1:**
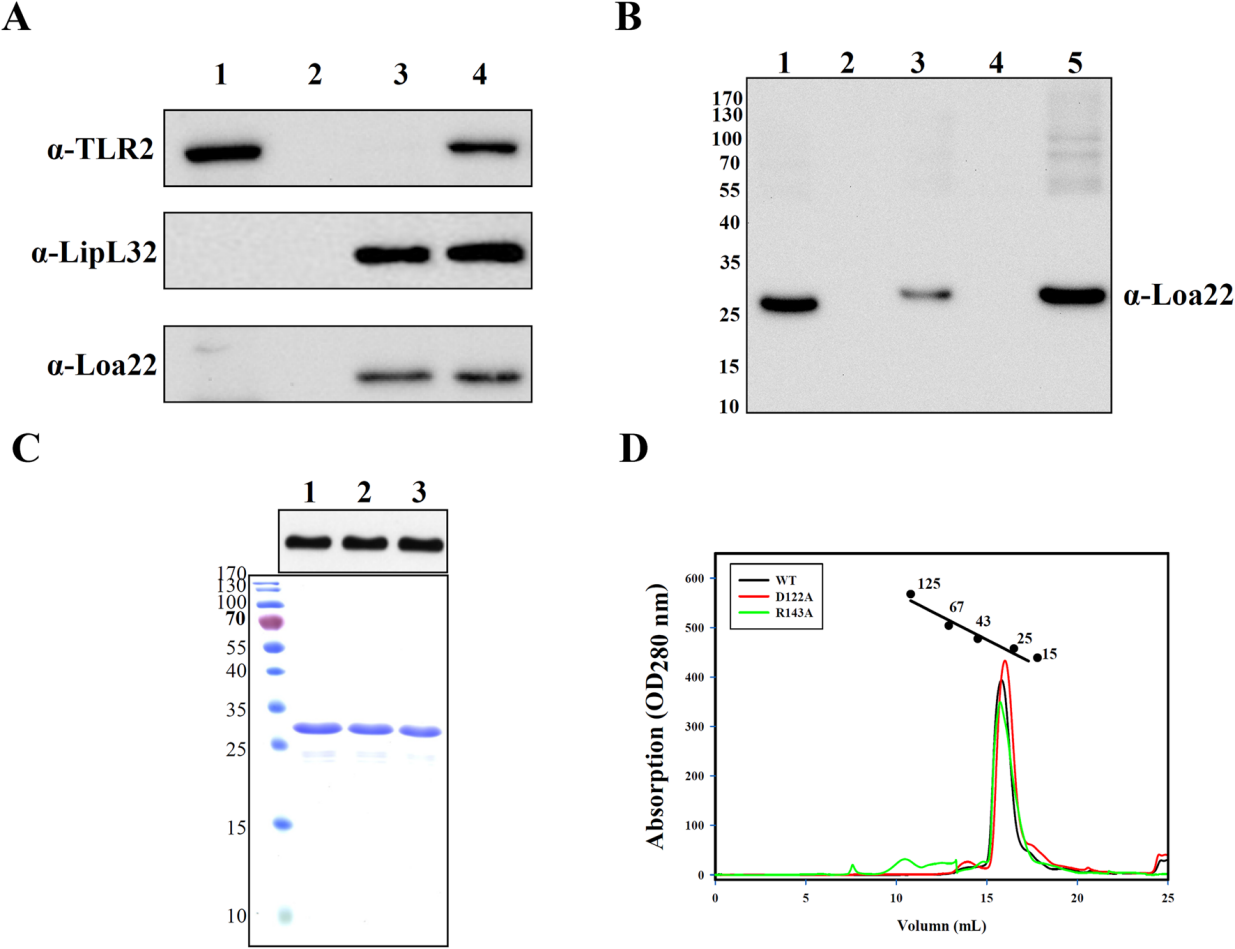
Characterization of TLR2 binding proteins from *Leptospira*. (**A**) Immuno-precipitant of purified TLR2 protein and Loa22 protein. Anti-TLR2, anti-LipL32 and anti-Loa22 antibodies were used to detect the presence of these proteins. Lane 1: anti-V5 antibody activated Protein A beads incubated with HEK293-TLR2 cell lysate; lane 2: anti-V5 antibody activated Protein A beads incubated with LOMP; lane 3: LOMP; lane 4: Co-IP of TLR2 and LOMP fractions. (**B**) The expression of Loa22 protein in different species and fractions of *Leptospira*. Recombinant Loa22 (rLoa22) was used as positive control (lane 1). Peptidoglycan from *L. santarosai* (LPGN) was also used to detect the presence of Loa22 protein (lane 2). LOMPs from different species of *Leptospires* were used to detect the presence or absence of Loa22 protein including pathogenic *L. interrogans* (lane 3), non-pathogenic *L. biflexa* (lane 4), and *L. santarosai* (lane 5). Rabbit polyclone anti-Loa22 antibody was use to recognize the protein. (**C**) Purification of the Loa22 and mutation variants. Wild type and mutation variants were analyzed by SDS-PAGE (lower panel) and Western blot (upper panel; recognized by anti 6XHis tag antibody) Lane 1: WT Loa222; lane 2; Loa22D122A; lane 3: Loa22R143A. (**D**) Size exclusion chromatography of purified Loa22 protein. The single peak of purified Loa22 protein with molecular mass about 22 kDa indicated that the protein was of uniform conformation. The standard markers were ferritin (440 kDa), β-galactosidase (125 kDa), albumin (67 kDa), ovalbumin (43 kDa), chymotrypsinogen A (25 kDa), and ribonucleaseA (15 kDa).

Upon infection by pathogens, innate immune responses are induced to defense against the infection on host cell surfaces. We attempted to identify the TLR2 ligands from pathogenic *L. santarosai* serovar Shermani and characterize the binding mechanisms of these virulence factors with TLR2. The human *TLR2* gene was sub-cloned from plasmid pUNO-TLR2 (Invivogen, San Diego, CA) and inserted into a lentivirus expression vector with a V5 tag at the C-terminus. The packaged virus particles were used to infect to HEK-293 T cells, and a stable clone was selected using blasticidin for over-expression of the full-length human TLR2 protein. HEK-293 T-TLR2 cells were used for full-length human TLR2 protein expression, and protein A-immobilized anti-V5 antibody was used for human TLR2 protein pull-down assays. After incubation of *Leptospira* outer membrane extractions with HEK-293 T-TLR2 cells for two hours, the cells were lysed and protein A-immobilized anti-V5 antibody was used to pull-down the TLR2 and binding candidates. The pull-down fractions were analyzed by western blot, and proteins were recognized with relative antibodies. Several TLR2 binding candidates were isolated and one of the positive controls, LipL32, was observed by anti-LipL32 antibody recognition (Fig. 1A)^22,23,25,26^. This result suggested that the method used for identification of TLR2-binding candidates searching and identification is suitable. An interesting TLR2 binding candidate, Loa22, was observed in the anti-Loa22 antibody recognition after co-immunoprecipitanting with TLR2. Western blot clearly demonstrated the interaction of Loa22 and TLR2 after co-immunoprecipitantion (Fig. 1A). Loa22 was present in pathogenic *Leptospires* but not in non-pathogenic *Leptospires*, indicating that Loa22 protein is probably a virulence factor (Fig. 1B)^10^. Loa22 is anchored to the outer membrane of pathogenic *Leptospires* and contains a large OmpA domain, known as a peptidoglycan-binding domain (Fig. S1A). Therefore, recombinant Loa22 (rLoa22) was constructed and expressed in *E. coli* to obtain purified rLoa22.

### Protein purification and mutagenesis

The Loa22 protein contains 195 amino acids, and domain prediction indicated the N-terminal signal peptide and C-terminal OmpA domain (Fig. S1A). Sequence alignments of Loa22 with other OmpA domain proteins (Pal protein from *E. coli* and OmpA protein from *A. baumannii*) indicated that sequence similarity was low; however, two important PGN-binding residues, Asp^122^ and Arg^143^, were highly conserved in these OmpA domain proteins (Fig. S1B). Therefore, these two residues were mutated to Ala using site-directed mutagenesis to generate D122A and R143A mutation variants. The rLoa22 protein was expressed in *E. coli* ClearColi BL21 (DE3) pLys (Lucigen, Middleton, WI) and further purified by Ni^2+^-NTA affinity column and size exclusion chromatography (Fig. 1C,D). In order to remove *E. coli* endotoxin contamination, the MonoQ column and polymyxin B resin were used to further remove endotoxin from purified rLoa22 protein. In the Limulus amebocyte lysate (LAL) assay, rLoa22 from *E. coli* Clearcoli contained negligible endotoxin and was suitable for inflammation assays (Fig. S1C)^22^.

### PGN binding assay

**Figure 2:**
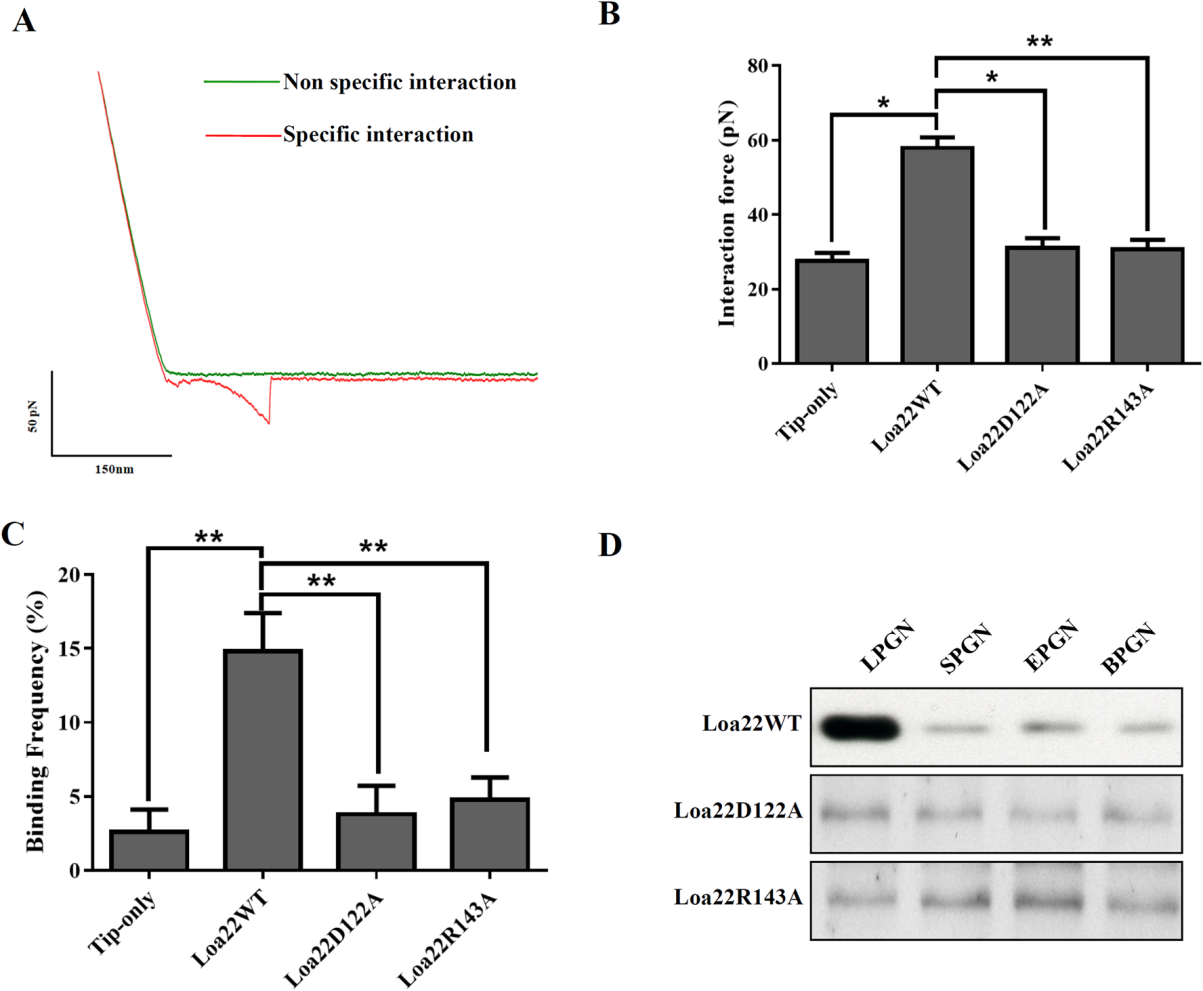
Rupture forces between rLoa22 and cell wall from *L. santarosai* surface as determined by smAFM. (**A**) The force-distance curves of AFM measurement. The tip-only were used to analyze the interaction to *L. santarosai* cell wall (green line). rLoa22 modified AFM tip were used to analyze the interaction to *L. santarosai* cell wall (red line). (**B**) The interaction forces of rLoa22 and its variants interacted with *Leptospira* cell wall. (**C**) The binding frequency of rLoa22WT and its variants interacted with *Leptospira* cell wall. rLoa22WT and mutation variants, D122A and R143A, were modified on AFM tip for rupture force measurements, respectively. (**D**) The PGN pull-down assay of rLoa22WT and its variants to PGN molecules including the LPGN and commercial available PGN molecules including *E. coli* (EPGN), *S. aureus* (SPGN), and *B. subtilis* (BPGN), respectively.*p* < 0.05;*p* < 0.01.

Loa22 is a lipoprotein with a C-terminal OmpA domain, which is speculated to bind the essential cell wall component, PGN. To verify PGN-binding activity of rLoa22, AFM was used to investigate the interaction between rLoa22 and LPGN. The *Leptospira* was immobilized on a mica surface and washed three times with PBS buffer containing 0.1% (w/v) Triton X-114 to remove the outer membrane and expose the PGN layer (Fig. S2A,B). The rLoa22-modified AFM tip was used to measure the affinity between rLoa22 and *Leptospira* cell wall. AFM force-distance curves were recorded to distinguish specific and non-specific interactions (Fig. 2A). The specific interaction force-distance curves were selected to analyze interactions between rLoa22 and LPGN. In contrast, the tip only was used to measure the *Leptospira* surface, as well as the rLoa22-modified AFM tip was used to measure affinity for the mica surface as a negative control. The interaction forces of the two controls were calculated as 26.3 ± 5.1 and 31.2 ± 4.7 pN, respectively (Figs. 2B and S2C-D). The interaction force between rLoa22WT and LPGN was calculated as 58.2 ± 5.6 pN (Figs. 2B and S2E). In addition, the binding frequency between rLoa22WT and LPGN was calculated as 15.8% as compared to that of mica surface as 2.1% (Fig. 2C). This result clearly demonstrated the LPGN binding activity of the purified rLoa22 protein. In addition, the rLoa22 mutation variants were coated on AFM tip for PGN binding activity measurement. As expected, the interaction forces and binding frequency of D122A and R143A variants displayed low levels of LPGN binding activity that indicated the two residues played vital roles in LPGN binding (Fig. 2B,C and Fig. S2F.G). The two rLoa22 variants exhibited gross impairment in LPGN binding ability as compared to rLoa22WT, suggesting their crucial roles in maintaining of LPGN binding by rLoa22. Besides, to investigate the PGN binding activity of rLoa22 to other different PGN molecules, commercially available PGN molecules including those from *E. coli* (EPGN), *S. aureus* (SPGN), and *B. subtilis* (BPGN) were selected for incubation with rLoa22 protein at 37 °C for 30 min. In addition, LPGN was isolated as described in Materials and Methods to test its affinity for rLoa22 protein. After three steps of centrifugation and washes, the pellets were subjected to SDS-PAGE and western blot analysis. The results indicated that rLoa22 protein showed relative high affinity for LPGN (Fig. 2C). An internal control of the purified LPGN was used to recognize by anti-Loa22 antibody and the result indicated that the purified LPGN contained no or less Loa22 in the purification process (Fig. 1B, lane 2). This result clear demonstrated the purified LPGN from pathogenic *Leptospira* contains no residual Loa22 protein. The mutated variants of rLoa22, D122A and R143A, showed low affinity for the four types of PGN molecules, indicating that the two residues are important for PGN binding. Taken together, these results demonstrated that PGN molecules from pathogenic *Leptospira* with high affinity for rLoa22, while other PGN molecules exhibited relatively low affinity for rLoa22 protein (Fig. 2C). Therefore, LPGN was selected for subsequent studies.

### rLoa22 Co-localizes with TLR2 on HEK293-TLR2 Cells

**Figure 3:**
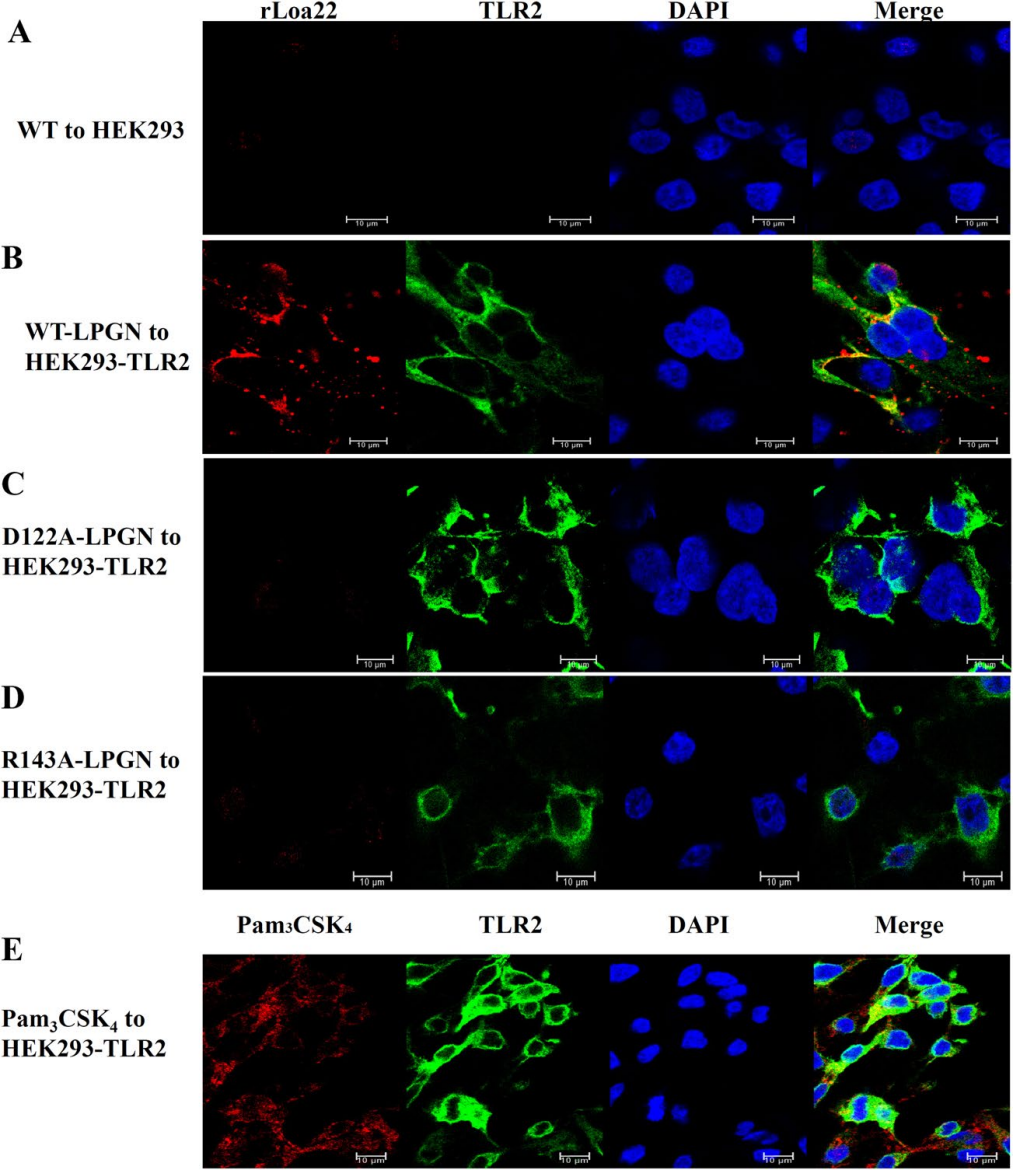
Loa22 co-localized with TLR2 on HEK293-TLR2 cells. HEK293-TLR2 cell were cultured to 70% confluence and serum free for 16 h before adding the stimulation agents (0.1 μg/ml). The cells were incubated with the stimulation agents for 4 h and then fixed to against with the anti-V5 antibody (1:5000), anti-Loa22 antibody (1:10,000). The relative Alex488 and Alex594 conjugated secondary antibodies were used to stained TLR2 and Loa22 proteins, respectively. (**A**) rLoa22WT was incubated with HEK293 cell. (**B**) rLoa22WT-LPGN was incubated with HEK293-TLR2 cell. (**C**) rLoa22D122A-LPGN was incubated with HEK293-TLR2 cell. (**D**) rLoa22R143A-LPGN was incubated with HEK293-TLR2 cell. (**E**) Pam_3_CSK_4_ Rhodamine was incubated with HEK293-TLR2 cell. The nucleus was stained by DAPI (blue) and TLR2 was stained by Alexa488 (green). Loa22 was stained by Alexa594 (red). The yellow color indicated that the two proteins were co-localized in HEK293-TLR2 cell.

Attachment of *Leptospira* outer membrane proteins to host cell membrane is the first step invading the host during *Leptospira* infection. Previous studies showed that the Loa22 is up-regulation when host infection induces high levels of antibody production in the infected patient’s serum^10,27^. However, the receptor on host cell membrane which recognizes Loa22 protein is still unknown and needs further investigation. The results mentioned above suggested that TLR2 is a possible receptor on host cell membranes for Loa22. In order to demonstrate the co-localization of rLoa22 and TLR2, purified rLoa22 protein and its variants were incubated with HEK293-TLR2 cells for 4 h and the cells were then washed, fixed, and incubated with conjugated antibodies for confocal microscopy analysis (Fig. 3). Besides, a positive control, Pam_3_CSK_4_ Rhodamine, was also used to incubate with HEK293-TLR2 cells to observe the colocolization behavior (Fig. 3E)^22^. rLoa22 and rTLR2 proteins were stained with rabbit polyclonal anti-Loa22 and mouse monoclonal anti-V5 primary antibodies follow by Alexa594 (red) conjugated anti-rabbit and Alexa488 (green) conjugated anti-mouse secondary antibodies, respectively. HEK293 cells lacking TLR2 expression were used as negative controls, with very little or no Alexa 488 fluorescence (Fig. 3A). Small amount of the Alexa 594 fluorescence revealed that the Loa22 bind to the HEK293 cell and this result is consisted with previous study that the Loa22 could bind the ECM molecules^12^. Co-localization of rLoa22WT-LPGN complexes and TLR2 receptors on HEK293-TLR2 cell was shown in Fig. 3B. The results indicated that TLR2 receptor and the rLoa22 protein were mostly present on the cell surface, with consistent partial localization in the cytosol. The merged colors in several portions indicated that the two proteins were co-localized on HEK293-TLR2 cells (Fig. 3B). In additions, the PGN molecules from other species including *E. coli* (EPGN), *S. aureus* (SPGN), and *B. subtilis* (BPGN) were used to test the ability to facilitate the interaction between rLoa22 and TLR2 on cell surface. The results indicated that the EPGN, SPGN, and BPGN could not mediate the interaction between rLoa22 and TLR2 (Fig. S3). In contrast, the two mutated variants reduced the cell binding ability, and the red color was absent in the confocal images (Fig. 3C,D). The results from confocal microscopy clearly showed rLoa22-LPGN complexes directly interacted with TLR2 on HEK293-TLR2 cell surface, while the mutated variants, rLoa22D122A-LPGN and rLoa22R143A-LPGN, of rLoa22 significantly decreased co-localization with TLR2 on the cell surface.

### Interaction between TLR2 and rLoa22-LPGN complexes

**Figure 4:**
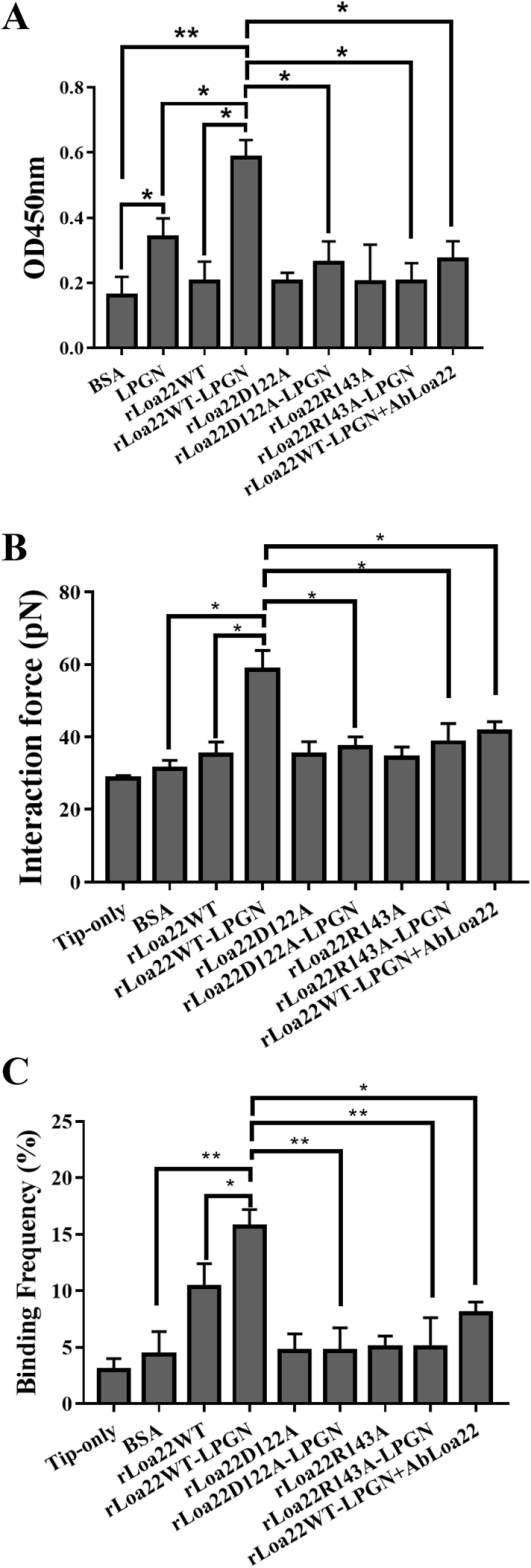
In vitro assay of the interaction between TLR2 and rLoa22-LPGN complexes. (**A**) ELISA assay of the interaction between TLR2 and rLoa22. (**B**) AFM force-distance curves of the interaction between TLR2 and rLoa22. (**C**) The binding frequency of the interaction between TLR2 and rLoa22. TLR2 interacted to rLoa22WT and mutation variants, rLoa22D122A and rLoa22R143A in the presence or absence of LPGN. BSA was used as negative control.*p* < 0.05;*p* < 0.01.

The purified TLR2 protein was used to measure the interaction between TLR2, rLoa22, and LPGN molecule. In ELISA assays, the LPGN molecule from pathogenic *Leptospira* showed high affinity to TLR2 protein and the affinity between LPGN and TLR2 significantly increased as compared to BSA control (Fig. 4A). The purified rLoa22WT slightly increased the affinity to TLR2. Interestingly, the rLoa22WT-LPGN complex significantly increased the affinity to TLR2 as compared to BSA control, LPGN, and rLoa22WT, respectively. The results indicated that LPGN molecule might played essential roles in rLoa22 and TLR2 affinity. In the presence or absence of LPGN, the affinities between TLR2 and the two mutation variants (rLoa22D122A and rLoa22R143A) showed similar to BSA control. The affinity between TLR2 and the mutation variants in complex with LPGN showed significantly decreased as compared to rLoa22WT-LPGN. The results demonstrated that two residues (Asp^122^ and Arg^143^) of rLoa22 were essential for the affinity between TLR2 and rLoa22-LPGN complex. In AFM measurements, the interaction force and binding frequency of rLoa22WT to TLR2 were slightly increased as compared to BSA control (Fig. 4B,C). Interestingly, the interaction force and binding frequency of rLoa22-LPGN complexes to TLR2 showed significantly increased as compared to BSA control (Fig. 4B,C). These results provided direct evidence that LPGN cooperated with rLoa22 to interact with TLR2. In order to identify which components of rLoa22-LPGN complex were responsible for interaction with TLR2, we used anti-rLoa22 antibody to block rLoa22 in rLoa22-LPGN complex and further treated with rTLR2 protein by using ELISA and AFM. The results indicated that anti-rLoa22 antibody efficiently reduced the affinities between rLoa22-LPGN complex and rTLR2. This result supported our hypothesis that Loa22 interacted with LPGN and induced conformational changes in the protein, which exposed the TLR2-binding domain of Loa22 to interact with TLR2. In addition, the interaction force and binding frequency of TLR2 to rLoa22 mutated variants (D122A and R143A) showed no significantly differences as compared to BSA control. It is not surprising that the rLoa22 mutation variants were loss of function variants that showed significantly decreased interaction force and binding frequency as compared to rLoa22WT-LPGN (Fig. 4B,C). Further addition of the LPGN molecules to these two variants could not raise the affinity to TLR2.

### rLoa22-LPGN complexes induced p38, ERK, and JNK dependent signaling

**Figure 5:**
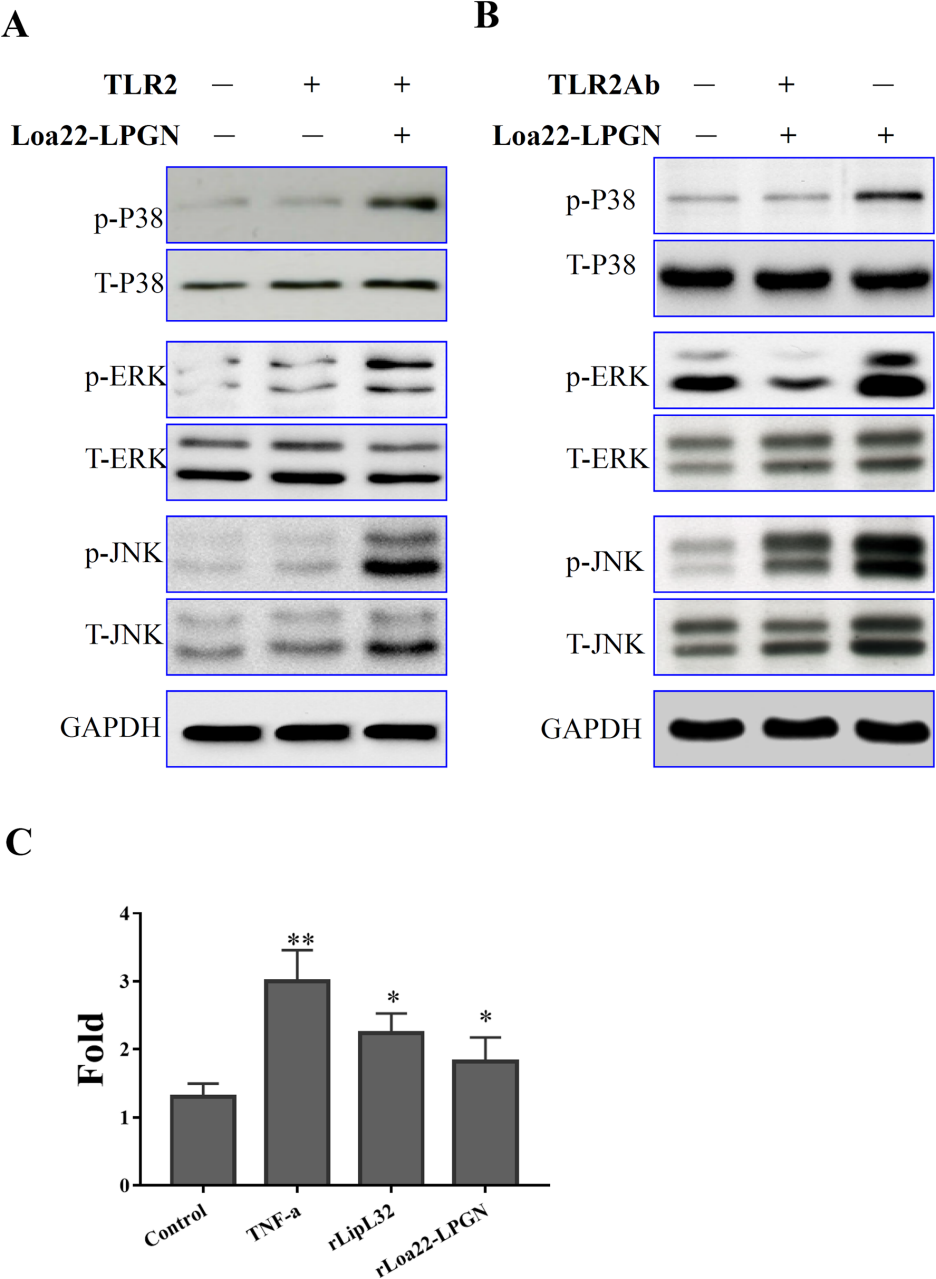
TLR2 downstream signaling cascades assays after rLoa22-LPGN complexes stimulation. HEK293-TLR2 cells were cultured to 70% confluence and serum free for 16 h before adding the stimulation agents (0.1 μg/ml). The cells were incubated with the stimulation agents for 1 h and collected for western blot analysis. (**A**) rLoa22-LPGN complexes stimulated HEK293 and HEK293-TLR2 cells for 1 h and downstream signaling cascades were assayed. (**B**) rLoa22-LPGN complexes stimulated HK2 cells in the presence and absence of TLR2 antibody and downstream signaling cascades were assayed. Anti-TLR2 antibody (1 μg/ml) was used to pretreated HK2 cell for 1 h before adding the rLoa22-LPGN complexes. (**C**) Luciferase assay of NF-κB in HEK293 cell. HEK293-TLR2 cell transfected with pNF-κB luc plasmid and NF-κB translocation activity was assayed by luciferase reporter assay. PBS was used as control and TNF-α was used as positive control. rLipL32 and rLoa22-LPGN were used to stimulate TLR2 downstream signaling cascades and promoted NF-κB translocated into nuclus.*p* < 0.05;*p* < 0.01.

*Leptospira* infection induces inflammatory responses through the TLR2-dependent pathway, and downstream signalings were therefore evaluated. The activation of the MAPK pathway was validated by western blot after treatment of HK2 and HEK293-TLR2 cells with rLoa22-LPGN complexes. The phosphorylation of MAPK pathway components including p38, ERK, and JNK was observed using their relevant antibodies. HK2 and HEK-293-TLR2 cells were cultured in serum free medium for 16 h before adding the stimulating agent, rLoa22-LPGN complexes to precise evaluations of cellular function. Different time points were tested to determine the maximum phosphorylation levels of p38, ERK, and JNK. The maximum phosphorylation levels occurred at 1 h after stimulation with rLoa22-LPGN complexes in serum-free HK2 and HEK-293-TLR2 cells. Stimulation of HEK293-TLR2 cells by rLoa22-LPGN complexes significantly increased the phosphorylation of p38, ERK, and JNK as compared to HEK-293 cells (Fig. 5A). For TLR2 antibody neutralization experiments, HK2 cells were pretreated with anti-TLR2 antibody (10 μg/ml) for 1 h, followed by adding of rLoa22-LPGN complexes for stimulation. Results in HK2 cells also revealed that cells pretreated with TLR2 antibody exhibited significantly decreased phosphorylation of p38, ERK, and JNK as compared to non-neutralized controls or non rLoa22-LPGN complexes stimulation controls (Fig. 5B). Besides, we also tested the NFκB nuclear translocation using luciferase assay. We transfected pNFκB luc plasmid (Stratagene, La Jolla, CA) into HEK293-TLR2 cell and further treated the cells with PBS (control), TNF-α, rLipL32, and rLoa22-LPGN to evaluate the NFκB nuclear translocation activity. The luciferase assays demonstrated that rLoa22-LPGN significantly increased the NFκB nuclear translocation activity as compare to PBS treatment (Fig. 5C). All these results suggested that rLoa22-LPGN complexes stimulate the production of inflammatory responses through p38, ERK, JNK, and NFκB signaling pathways.

### Inflammatory responses induces by rLoa22-LPGN complexes

**Figure 6:**
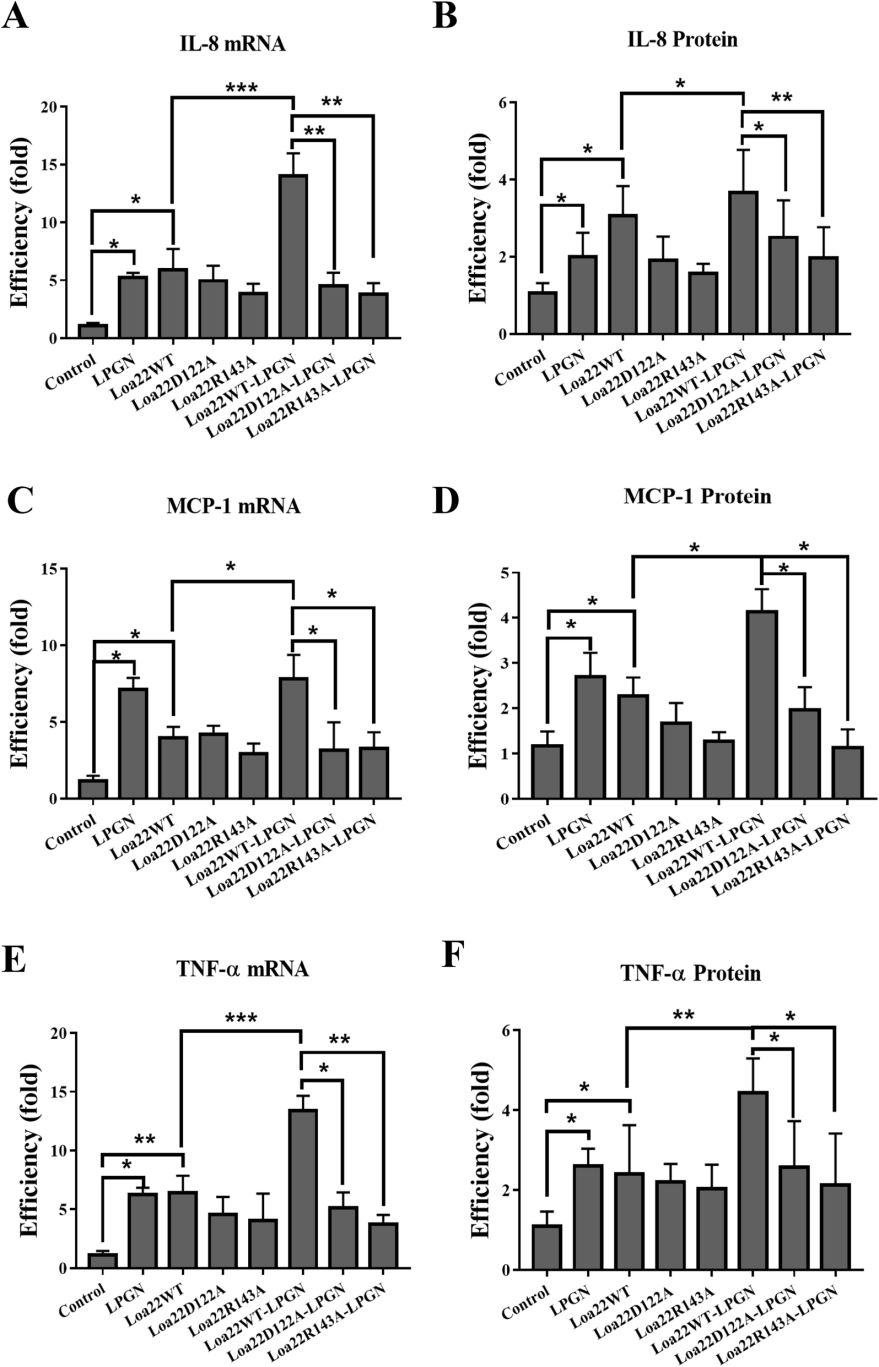
Inflammatory responses induced by rLoa22-LPGN in HEK293-TLR2 cells. HEK293-TLR2 cells were cultured to 70% confluence and changed to serum free conditions for 16 h. The stimulation agents (0.1 μg/ml) were added to stimulate the downstream inflammatory responses from TLR2 signaling such as *CXCL8/IL8*, *hCCL2/MCP-1*, and *hTNF-α*. (**A**) Stimulation of the expression of *CXCL8/IL8* mRNA. (**B**) Stimulation of the expression of CXCL8/IL8 protein. (**C**) Stimulation of the expression of *hCCL2/MCP-1* mRNA. (**D**) Stimulation of the expression of hCCL2/MCP-1 protein (**E**) Stimulation of the expression of *hTNF-α* mRNA. (**F**) Stimulation of the expression of hTNF-α protein. Inflammatory responses stimulated by Loa22 and its variants (rLoa22WT, rLoa22D122A, and rLoa22R143A) in the presence or absence of LPGN. The results of mRNA levels in different genes are displayed as the transcript levels of the analyzed genes relative to GAPDH (glyceraldehyde-3-phosphate dehydrogenase) transcript level. The secretion cytokines were measured by ELISA.*p* < 0.05;*p* < 0.01;*p* < 0.001.

Recognition of bacterial components by host TLRs initiates signaling cascades that stimulate nuclear transcription factor *κ*B (NF-κB) and mitogen-activated protein kinases (MAPKs), and induces expression of chemokines and cytokines^25,26^. rLoa22 was expressed in *E. coli* ClearColi BL21 (DE3) pLys that contained low levels of endotoxin and was further purified by Ni^2+^-columns, MonoQ, and polymyxin to remove the contaminating endotoxin (Fig. S1C). The mRNA and protein expression levels of *CXCL8/IL8*, *hCCL2/MCP-1*, and *hTNF-α* were measured to investigate the role of rLoa22 HEK293-TLR2 cells. Two hours after incubating with the stimulation agents, the HEK293-TLR2 cells were collected for mRNA analysis. In the ELISA assays, HEK293-TLR2 cells were incubated with the stimulation agents for 8 h, the supernatants were collected for cytokines measurements. Purified LipL32 protein was used as the positive control, and PBS buffer alone was served as negative control^23^. In HEK293-TLR2 cells, LipL32 and Loa22WT significantly increased the mRNA and protein expression of CXCL8/IL8, hCCL2/MCP-1, and hTNF-α as compared to the PBS control (Fig. S4). Further confirming the inflammatory cytokines induced by recombinant Loa22 protein, the Loa22 was heat-treated (100 °C, 30 min) and digested with proteinase K (20 µg/ml at 63 °C for 18 h), and the results revealed that the denatured and digested rLoa22 protein significantly decreased the mRNA and protein expression of CXCL8/IL8, hCCL2/MCP-1, and hTNF-α as compared to that of Loa22WT (Fig. S4). The absence of stimulatory effects after heat and proteinase K treatments further demonstrated that the Loa22WT provoked the inflammatory responses. Besides, LPGN and Loa22WT significantly increased the mRNA and protein expression of CXCL8/IL8, hCCL2/MCP-1, and hTNF-α as compared to the PBS control (Fig. 6). Furthermore, the rLoa22-LPGN complexes significantly increased mRNA and protein expression levels of CXCL8/IL8, hCCL2/MCP-1, and hTNF-α as compared to that of Loa22WT (Fig. 6)^22,25^. We further investigated the roles of LPGN and the mutated variants of rLoa22 in the stimulation of inflammatory responses. The rLoa22D122A and rLoa22R143A mutated variants with low affinity to LPGN showed relative low ability to stimulate the mRNA and protein expression levels of CXCL8/IL8, hCCL2/MCP-1, and hTNF-α (Fig. 6). The mutation variants (Loa22D122A and Loa22R143A) in complex with LPGN significantly decreased the mRNA and protein expression levels of CXCL8/IL8, hCCL2/MCP-1, and hTNF-α as compared to that of rLoa22WT-LPGN complexes. The results further demonstrated that the LPGN cooperated with rLoa22 to interact with TLR2 and stimulated inflammatory responses. In order to identify the inflammation induced by Loa22-LPGN in primary human cells, human monocytic cell line (THP-1), was used to measure the cytokines production under the stimulation of Loa22-LPGN. THP-1 was cultured to 2 × 10^7^/well and induced the differentiation into macrophages using phorbol-12-myristate-13-acetate (PMA). In THP-1 cells, rLoa22-LPGN significantly increased the protein expression of hCXCL8/IL8, hCCL2/MCP-1, and hTNF-α that indicated the rLoa22-LPGN stimulated cytokines production in primary human cells and transfected cell lines of macrophage (Fig. S5).

**Figure 7:**
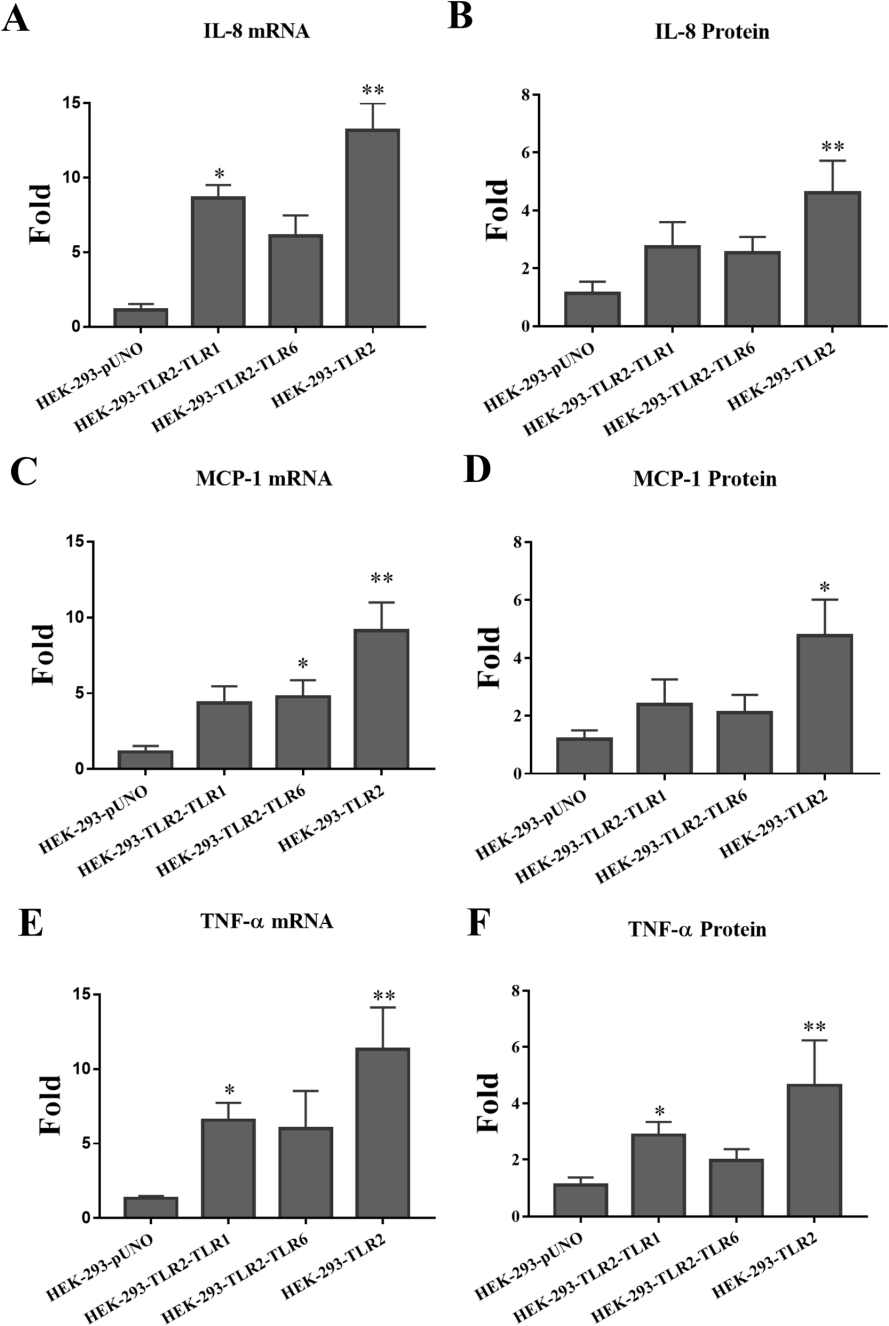
Inflammatory responses induced by rLoa22-LPGN in transient transfection HEK293 cells. Several plasmids (TLR2-TLR1, TLR2-TLR1, and TLR2 only) were transiently transfected into HEK-293 cell and the inflammatory responses induced by rLoa22-LPGN complex were assayed including hCXCL8/IL8, hCCL2/MCP-1, and hTNF-α. (**A**) Stimulation of the expression of *CXCL8/IL8* mRNA. (**B**) Stimulation of the expression of CXCL8/IL8 protein. (**C**) Stimulation of the expression of *hCCL2/MCP-1* mRNA. (**D**) Stimulation of the expression of hCCL2/MCP-1 protein. (**E**) Stimulation of the expression of *hTNF-α* mRNA. (F) Stimulation of the expression of hTNF-α protein. HEK-293-pUNO was used as mock control. *p* < 0.05;*p* < 0.01.

Previous studies suggested that TLR2 preferred to form heterodimer with either TLR1 or 6 depended on different antigens stimulation^18,28^. In order to identify which heterodimer is important to therLoa22-LPGN complex, we co-transfected of TLR2-TLR1 and TLR2-TLR6 into HEK293 cell and stimulated these cells with rLoa22-LPGN complexes to identify which TLRs cooperated with TLR2 in responses of rLoa22-LPGN complexes. The transfection efficiencies of these genes into HEK-293 cells were similar (Fig. S6). The inflammatory responses induced by rLoa22-LPGN complex were assayed including hCXCL8/IL8, hCCL2/MCP-1, and hTNF-α (Fig. 7). The expression level of mRNA was measured by real time PCR and the expression level of protein was measured by ELISA to identify the simulation of rLoa22-LPGN complex in the transfected HEK-293 cells. The results indicated that rLoa20-LPGN stimulated highest levels of cytokines expression mainly through TLR2 (Fig. 7). In the stimulation of IL-8, the rLoa22-LPGN complex also significantly increased mRNA expression in HEK-293-TLR2-TLR1 cells (Fig. 7A). In the stimulation of MCP-1, the rLoa22-LPGN complex significantly increased mRNA expression in HEK-293-TLR2-TLR6 cells (Fig. 7B). In the stimulation of TNF-α, the rLoa22-LPGN complex significantly increased mRNA and protein expression in HEK-293-TLR2-TLR1 cells (Fig. 7C,F).

## Discussion

In *Leptospira*, Loa22 has been shown as an essential virulence factor, in that deletion of the Loa22 from pathogenic *Leptospira* attenuates toxicity, whereas the re-expression the gene in *Leptospira* restores the virulence^11^. *Leptospira biflexa* serovar Patoc is non pathogenic strain that contains a Loa22-like gene (WP_012390072.1) but the expression of this gene seemed seemed to be downregulated that the protein level can hardly be detected (Fig. 1B)^10^. However, the pathogenic mechanisms and the vital domains of Loa22 are still unclear. In this study, we used pull-down assays to demonstrate the interactions between TLR2 and Loa22 from *Leptospira* outer membrane extractions. One of the positive controls of this pull-down assay was LipL32, which has been proven as a TLR2 binding protein in pathogenic *Leptospira*^22,25,26^. Interestingly, domain prediction of Loa22 protein suggested an OmpA-like domain and was speculated to interact with PGN. Therefore, PGN molecules were used to investigate the affinity with Loa22. However, PGN molecules from *E. coli* (EPGN), *S. aureus* (SPGN), and *B. subtilis* (BPGN) showed low affinity for Loa22. PGN molecules from pathogenic *Leptospira* (LPGN) are the only PGN molecule that showed relative high affinity for Loa22 (Fig. 2D). These results suggested that the structure and composition of LPGN were different from those of EPGN, SPGN, and BPGN, and required further investigation. Combined with AFM force-distance curve studies and site-directed mutagenesis demonstrated that Loa22 directly interacted with LPGN on bacterial surfaces and two vital residues were involved in the interaction between Loa22 and LPGN (Fig. 2). Furthermore, Loa22-LPGN complexes were observed to co-localize with TLR2 on HEK293-TLR2 cell surfaces (Fig. 3). The two mutation variants with low or no affinity for LPGN also revealed that the co-localization behaviors to TLR2 on HEK293-TLR2 cell surface were attenuated (Fig. 3). In addition, the interaction forces and binding frequencies between rLoa22 and TLR2 showed no significant difference as compared to BSA control whereas the interaction forces and binding frequencies between rLoa22-LPGN complexes and TLR2 showed significantly increased. The rLoa22 mutation variants also decreased the affinity for TLR2 in the absence or presence of LPGN as compared to rLoa22WT-LPGN complexes (Fig. 4). We suggested two possibilities models concerning the relationships between rLoa22, LPGN, and TLR2. Firstly, Loa22 could not directly bind to TLR2; rather Loa22 used LPGN to interact with TLR2. According to this model, the concept of defining PGN as the TLR2 ligand is controversial, and whether PGN from pathogenic *Leptospira* as the TLR2 ligand is still unclear. The unique PGN from pathogenic *Leptospira* also showed higher affinities for Loa22 as compared to those from *E. coli*, *S. aureus*, and *B. subtilis* (Fig. 2D). *Leptospira* is an idiographic bacterium that differs from other pathogens and its composition and reaction to host cells required further elucidation. Secondly, Loa22 interacted with LPGN and induced conformational changes in the protein, which exposed the TLR2-binding domain of Loa22 to interact with TLR2. The second model is more likely, wherein LPGN induces conformational changes in Loa22 and therefore triggers the interaction between Loa22-LPGN and TLR2. Evidence for this model is that the Loa22 mutation variants exhibited low affinity for LPGN and decreased the affinity for TLR2. Besides, anti-rLoa22 antibody was used to neutralize the rLoa22-LPGN complex and the result showed that the affinity between TLR2 and rLoa22-LPGN were attenuated. This study provides further indication of a role of LPGN participating in mediating *Leptospira* recognition by the innate immune system and as a potential target for anti-*Leptospira* treatment.

It has been reported that TLR2 interacts with PGN and induces inflammatory responses^24^. In this study, rLoa22 was demonstrated to interact with LPGN through the two vital residues, and consequently interacted with TLR2 to induce downstream signals and cytokines production. Downstream signals induced by rLoa22-LPGN complexes were explored in HEK293 and HK2 cells. MAPK signal transduction pathway components, including p38, ERK, and JNK, were obviously stimulated. Previous studies have reported that HEK293 cell express no TLR2 on cell surfaces, and we constructed HEK293-TLR2 cells to express TLR2^22^. HEK293 cells were used as negative controls. rLoa22-LPGN complexes significantly up-regulated the phosphorylation levels of p38, ERK, and JNK in HEK293-TLR2 cells, but not in HEK293 cells (Fig. 5A). In HK2 cells, adding anti-TLR2 antibody for neutralization procedure blocked the interaction between rLoa22-LPGN and TLR2, and therefore alleviated the activation of the MAPK pathway (Fig. 5B). In the NF-κB nuclear translocation assay, rLoa22-LPGN significantly increased the NF-κB nuclear translocation activity as compare to PBS treatment, similar to the positive control TNF-α (Fig. 5C).

The inflammatory responses provoked by rLoa22 in HEK293-TLR2 cells were used to measure the toxicity of this essential virulence factor. Previous studies have shown that *Leptospira* outer membrane proteins induced expression of nitric oxide, MCP-1, and TNF-*α* in cells^29^. Tian et al*.* also demonstrated that outer membrane proteins of *L. santarosai* Shermani increased collagen and TGF-β in renal proximal tubular cells^2^. These data suggested that proinflammatory cytokines production might be involved in tubulointerstitial nephritis caused by *L. santarosai* Shermani infection through its outer membrane components. The recombinant protein method is the best way to investigate structural and functional relationships. However, endotoxin contamination from the recombinant protein can interfere with the intrinsic inflammatory properties of the innate immunity system. The endotoxin-free expression system provides the solution to overcome this problem. The ClearColi BL21(DE3) expression system was used to produce endotoxin free rLoa22 and its variants, and the yield of protein expressed was similar to that expressed in the BL21(DE3) expression system^30^. In addition, anion-exchange chromatography (Mono-Q) and polymyxin B resin were further used to remove contaminating endotoxin from purified rLoa22 and its variants. The LAL assay was used to confirm that the endotoxin was removed after the purification processes (Fig. S1C). In terms of inflammatory responses, rLoa22WT protein showed low ability to induce inflammatory responses, whereas when rLoa22 combined with LPGN induced an increased inflammatory response, similar to the major outer membrane LipL32 protein^22^. In fact, LPGN could significantly stimulate the expression of CXCL8/IL8, hCCL2/MCP-1, and hTNF-α (Fig. 6)*.* Muller-Anstett et al*.* reported that SPGN co-localized with TLR2 and stimulated innate immune responses^24^. The inflammatory responses induced by rLoa22WT-LPGN complex showed significantly increased as compared to that of Loa22WT (Fig. 6). These results demonstrated that rLoa22-LPGN complexes effectively stimulated immune responses much more than LGPN alone, and further demonstrated that the inflammatory responses were increased from Loa22-LPGN complex. On the other hand, the rLoa22 mutation variants also reduced the ability to induce inflammatory responses in the presence or absence of LPGN as compared to that of rLoa22WT-LPGN complex (Fig. 6). These results supported our hypothesis that LPGN bind rLoa22 and induced conformational changes to interact with TLR2 on cell surfaces. Furthermore, TLR2 forms heterodimers with TLR1 or TLR6 to recognize different pathogen antigens and rLoa22-LPGN was also used to interact with different TLR pairs. In this regard, when HEK-293 cells were co-transfected with TLR2-TLR1, TLR2-TLR6, or TLR2 only plasmid DNA into the HEK-293 cell and assayed the inflammatory responses induced by Loa22-LPGN and the results indicated that rLoa20-LPGN complex stimulated cytokines expression mainly through TLR2 (Fig. 7). TLR2 might formed homodimer on cell surface and recognized the rLoa20-LPGN complex to induce the downstream signals^6^.

In summary, our study demonstrated that Loa22 protein is a PGN binding protein and the PGN from *Leptospira* showed high affinity for Loa22. The LPGN binding activity of Loa22 is an important biological reaction to maintain cell wall stability and to protect against immune attack when infecting host cells. We further demonstrated that two key residues within the OmpA domain, Asp^122^ and Arg^143^, were involved in the affinity of Loa22 for LPGN. The interaction of Loa22 and TLR2 was explored by ELISA and AFM and the role of LPGN was verified to mediate Loa22 to interact with TLR2. Finally, Loa22 was proposed to interact with LPGN through two vital residues, and subsequently interacts with TLR2. This study showed that LPGN in *Leptospira* mediates interactions between Loa22 and TLR2 and increases downstream signals to trigger inflammatory responses. Interactions between Loa22-LPGN-TLR2 reveal a novel binding mechanism for the innate immune system and infection induced by *Leptospira*.

## Methods

### Cell and bacterial culture

HK-2 (ATCC number CRL-2190), HEK293 cells (ATCC CRL-1573), and THP-1 (ATCC number TIB-202) were obtained from the American Type Culture Collection (ATCC; Maryland, USA) and cultured in the culture medium mentioned previously^22,25,31^. Cells were grown in an incubator at 37 °C and an humidified atmosphere of 5% CO_2_. All experiments were performed under serum free conditions to avoid the influence of serum on cell function and investigated events. *L. santarosai* serovar Shermani str. LT821 (ATCC number 43286; pathogenic species), *L. interrogans* serovar Copenhageni Fiocruz L1-130 (ATCC number BAA-1198; pathogenic species), and *L. biflexa* serovar Patoc (ATCC number 23582; nonpathogenic species) purchased from the ATCC (Manassas, VA) were used in this study. These *Leptospira* were propagated at 28 °C under aerobic conditions in the medium mentioned previously^6,32^. Bacterial densities were counted with a CASY-Model TT cell counter and analyzer (Roche Innovatis AG, Casy-Technologh, Reutlingen, Germany).

### TLR2 over-expressed cell line

The p-TLR2-Lenti plasmid for over-expressed TLR2 in HEK-293 cell was constructed as mentioned previously^22^. The p-TLR2-Lenti plasmid was transfected into HEK-293 cells with ViraPower packaging mix to generate the lentivirus according to manufacturer’s protocol. HEK-293 cells were transfected with the lentivirus and stable cell lines were generated^22^. Cells were named as HEK293-TLR2 cells and collected for following experiments.

### Transient transfection and luciferase assay in HEK293 cells

HEK293 cells were transfected with pUNO, pUNO-TLR2, pUNO-TLR2 plus pUNO-TLR1, and pUNO-TLR2 plus pUNO-TLR6 (Invivogen) to construct the control, HEK293-TLR2, HEK293-TLR2-TLR1, and HEK293-TLR2-TLR6 cells, respectively. The Lipofectamine 2000 was used for transient transfection of these genes into HEK-293 cells according to the transfection protocol (Life Technologies Inc., Carlsbad, CA). The transfection efficiencies of these genes into HEK293 cells were confirmed by flow cytometry. The relative antibodies for TLR1 (#12–4714-81; Thermo Fisher Scientific, Waltham, MA), TLR2 (#11–9922-42; Thermo Fisher Scientific, Waltham, MA), and TLR6 (#MA5-16,177; Thermo Fisher Scientific, Waltham, MA) were used to stain the transfected HEK293 cells. In the reporter assay, HEK293 cells were transfected with pNFκB-Luc plasmid (Stratagene, La Jolla, CA) and Dual-Luciferase Reporter (DLR) assay system was used to assay the NFκB translocation activity according to the manufacturer’s protocol.

### *Leptospira* outer membrane extraction

*L. santarosai* serovar Shermani, *L. interrogans* serovar Copenhageni, and *L. biflexa* serovar Patoc (used as a negative control) were grown in 10% Ellinghausen McCullough Johnson Harris (EMJH) *Leptospiral* enrichment medium (Detroit, MI). These *Leptospires* were cultured for 5–7 days at 28 °C until they reached a cell density of 10^8^/ml as previously described^6^. The *Leptospira* outer membrane extractions were extracted with 1% Triton X-114 using the extraction protocol mentioned previously^33^. Briefly, the *Leptospires* were centrifuged and washed in phosphate-buffered saline (PBS) supplemented with 5 mM MgCl_2_ and then extracted at 4 °C in the presence of 1% Triton X-114 (protein grade), 10 mM Tris (pH 8.0), 1 mM phenylmethylsulfonyl fluoride (PMSF), 1 mM iodoacetamide, and 10 mM ethylenediaminetetraacetic acid. Insoluble material was removed by centrifugation at 17,000 *x*g for 10 min. Phase separation was conducted by warming the supernatant to 37 °C and subjecting it to centrifugation for 10 min at 2000 *x*g. The detergent and aqueous phases were separated and precipitated with acetone and lyophilized. The extracts were further dissolved in sterile H_2_O, filtered through 0.22 µm membrane filters, and stored at -80 °C until use.

### Pull-down assay

HEK293-TLR2 cell was used to express the human TLR2 protein for pull-down assay. After 48 h cell culture, HEK293-TLR2 cells were incubated with LOMP (10 μg/ml) for 4 h, followed by three times of PBS washes and centrifugation to remove unbound LOMP. The cells were lysed with Cell Lysis Buffer (Abcam; ab152163) and the supernatant were incubated with mouse anti-V5-tag antibody or mouse IgG (negative control) at 4 °C overnight, and then incubated with protein A–agarose beads (Roche) for 4 h. The beads were washed four times with lysis buffer, and the obtained samples were analyzed by 12% (w/v) SDS-PAGE.

### DNA construction and mutagenesis

The *loa22* gene (LSS_RS00795; 591 bp) was cloned from pathogenic *L*. santarosai serovar Shermani genomic DNA using pfu-Turbo DNA polymerase (Stratagene, La Jolla, CA)^23^. The primers used for *loa22* gene construction were listed in Table 1. After restriction enzyme double digestion, the PCR product was individually inserted into the expression vector pRSET (Invitrogen, Groningen, Netherlands). The point mutation of *loa22* variants were obtained by using Q5 Site-Directed Mutagenesis Kit (NEB, Ipswich, MA) with their relevant primers listed in Table 1. The plasmid DNA was verified by DNA sequencing.

**Table 1:** Primers used in this study.

Genes	Forward (5′ → 3′)	Reverse (5′ → 3′)
TNF-α	ATGAGCACAGAAAGCATGATCCGC	CCAAAGTAGACCTGCCCGGACTC
CXCL8/IL-8	ATGACTTCCAAGCTGGCCGTGGCT	TCTCAGCCCTCTTCAAAAACTTCTC
CCL2/MCP-1	CCGCTGTTATAACTTCACC	ACATCCCAGGGGTAGAACTG
hTLR2	CGACGCGTAGCATGCCACATAC	GCACGCGTGGACTTTATCGCA
Loa22WT	GGATCCATGGTCAAAAAAATTTTG	AAGCTTTTATTGTTGTGGAGC
Loa22D122A	CCGGACACACCGCTGCTATCGGACCC	GGGTCCGATAGCAGCGGTGTGTCCGG
Loa22R143A	CTTTTATTCCGAACTTGCTGCAAATGCCG	CGGCATTTGCAGCAAGTTCGGAATAAAAG

### Bioinformation analysis

Multiple sequence alignment of Loa22 protein from *L. shermani* and AbOmpA and Pal proteins from *A. baumannii* and *E. coli* were performed with TEXSHADE program^34^. The SMART http://smart.embl-heidlbergde/ and LipoP http://www.cbs.dtu.dk/services/LipoP/ web servers were used to search predicted functional and structural domains of Loa22^35,36^.

### Expression and purification of Loa22 and antibody preparation

The DNA constructs of Loa22 were individually transformed into expression host cell *E.coli* ClearColi BL21 (DE3) pLys (Lucigen, Middleton, WI). Loa22 and its variants were expressed and purified by using the methods mentioned previously^22,25,26^. The imidazole was removed by dialysis before assays. rLoa22 antigens were also used to induce the production of the polyclonal antibodies (anti-Loa22 antibody) by customized product (ABclonal Inc., MA, USA).

### Confocal microscopy

HEK293 and HEK293-TLR2 cells were fixed, permeabilized and incubated with appropriate primary and secondary antibodies: mouse monoclonal anti-TLR2 antibody (eBioscience, San Diego, CA), rabbit polyclonal anti-Loa22 and anti-LipL32 antibodies (ABclonal Inc., MA, USA)^22^. The secondary antibodies for confocal laser scanning microscopy were Alexa594 conjugated anti-rabbit and Alexa488 conjugated anti-mouse secondary antibodies (Research Diagnostics, Inc.). Cells were imaged by confocal laser scanning microscopy (TCS-SP8-X, Leica, Wetzlar, Germany).

### RNA extraction and real-time PCR

HEK293 and HEK293-TLR2 cells were incubated at 37 °C in a humidified atmosphere of 5% (v/v) CO_2_ for 70% confluence as previously described^6^. Cells were shifted to a serum-free medium for 24 h before adding the stimulation agents to the cell culture medium. Total RNA was extracted according to the guanidinium thiocyanate/phenol/chloroform method (Cinna/Biotecx Laboratories International Inc., Friendswood, TX)^6,37^. Real-time PCR was executed on the basis of the manufacturer's instructions using an ABI Prism 7700 with SYBR green I as a double-stranded DNA-specific dye (PE-Applied Biosystems, Cheshire, Great Britain). The primers of *hTNF-α*, *hCXCL8/IL-8*, and *hCCL2/MCP-1* were shown in Table 1 and constructed to be compatible with a single reverse transcription-PCR thermal profile (95 °C for 10 min, 40 cycles at 95 °C for 30 s, 60 °C for 1 min, and 72 °C for 3 min). The accumulation of the PCR product was recorded in real time (PE-Applied Biosystems). The results of mRNA levels in different genes are displayed as the transcript levels of the analyzed genes relative to GAPDH (glyceraldehyde-3-phosphate dehydrogenase) transcript level.

### *Leptospira* PGN preparation

*L. santarosai* serovar Shermani PGN was extracted according to the procedure of previous report^38^. Briefly, 2 L of *Leptospira* culture were harvested by centrifugation at 10,000 *xg* for 30 min, washed three times with PBS buffer, and resuspended in 100 ml 1% (w/v) SDS in distilled water. The suspension was gently shaken at 37 °C for 18 h and then centrifuged at 110,000 *xg* for 60 min. After a second treatment with 1% (w/v) SDS the pellet was washed three times with 6 M urea. The pellet was further resuspended in 100 ml distilled water and collected by centrifugation at 110,000 *xg* to remove the urea. The pellet was suspended in 10 ml 10 mM Tris–HCl buffer (pH 7.4) containing 0.1 mg/ml trypsin and incubated at 37 °C for 18 h. The pellet was collected by centrifugation at 110,000 *xg* for 90 min and suspended in 10 ml 10 mM Tris–HCl buffer (pH 7.4) containing 0.1 mg/ml pronase (Sigma) for incubation at 37 °C for 18 h. After digestion at 37 °C for 18 h the pellet was recovered by centrifugation at 110,000 *xg* for 90 min and then lyophilized. The amount of PGN extracted was measured of the dry weight of pellet and resuspended in distilled water for 1 mg/ml and stock at -80 °C until use.

### Enzyme-linked immunosorbent assay (ELISA)

The ELISA methods were used to investigate the interaction between Loa22 and TLR2 and the processes of this method was performed according to previously reports with minor modifications^25,39^. Briefly, rTLR2 (1 μg) protein was coated on ELISA plates and Loa22 and its variants (2 μM) were used to interact with rTLR2. Anti-Loa22 antibody (1:10,000 dilution) was used to detect the amount of rLoa22 protein. The binding data were analyzed via SigmaPlot 10.0 program by fitting to the optimal equation^40^.

### AFM Measurement and Analysis

The AFM cantilever tips were functionalized according to previous methods to modify the rLoa22 protein and its variants^25,26,41^. The mica surface was modified for deposition of rTLR2 protein according to previous report^42,43^. The unbound proteins were removed and tips and mica were stored at 4 °C until use. A commercial atomic force microscope (Nanoscope III, Digital Instruments, Santa Barbara, CA) with a J type scanner was employed throughout this study. The distance-force curves and force parameters were obtained according to the methods previously described^25,41^. All the measurements described above were performed with modified tips and showed repeatedly similar results.

### Statistical analysis

All experiments were performed at least three independent processes and the variables are expressed as mean ± SEM and compared by using Student’s t-test or one-way ANOVA. *p* values < 0.05 are considered statistically significant. All analyses were performed using the Graphpad Prism 5.1 (Graphpad, La Jolla, CA).

## Supplementary information

Supplementary Information.
