# Supplementary material for: Peptidoglycan mediates Leptospira outer membrane protein Loa22 to toll-like receptor 2 for inflammatory interaction: a novel innate immune recognition
Source: Sci Rep. 2021 Jan 13;11:1064. doi: 10.1038/s41598-020-79662-8 (PMC8115183; doi:10.1038/s41598-020-79662-8)

**Supplementary Figures**

**Peptidoglycan Mediates *Leptospira* Outer Membrane Protein Loa22 to Toll-Like Receptor 2 for Inflammatory Interaction: A Novel Innate Immune Recognition**

Shen-Hsing Hsu^1^, Ming-Yang Chang^1^, Shih-Ming Lin^2^, Yi-Ching Ko^1^, Li-Feng Chou^1^, Ya-Chung Tian^1^, Cheng-Chieh Hung^1*^, Chih-Wei Yang^1*^

^1^Kidney Research Center, Chang Gung Memorial Hospital, Linkou, Taoyuan, Taiwan; ^2^College of Medicine, Chang Gung University, Taoyuan, Taiwan

^2^Department of Biotechnology and Bioindustry Sciences, National Cheng Kung University, Tainan, 70101, Taiwan.

*Corresponding author: Chih-Wei Yang and Cheng-Chieh Hung, Department of Nephrology, Kidney Research Center, Chang Gung Memorial Hospital, Chang Gung University College of Medicine, 5 Fu-Shing St. Taoyuan 333, Taiwan, ROC. To Chih-Wei Yang Tel/Fax: +886-3-3281200-2506/+886-3-3282173; E-mail: [cwyang@ms1.hinet.net](mailto:cwyang@ms1.hinet.net). To Cheng-Chieh Hung +886-3-3281200-2506/+886-3-3285095; E-mail: [cchung@cgmh.org.tw](mailto:cchung@cgmh.org.tw)

Running title : LPGN mediates Loa22 and TLR2 interaction

**The supplementary file contains Figure S1- S6 and the legends**

**Supplementary Figure Legends**

**Supplementary Figure 1. Bioinformatic analysis and characterization of Loa22 from pathogenic *Leptospira*.** (A) Domains prediction of Loa22. Loa22 contains 195 amino acids and the domains preditions revealed that the N terminal contains signal peptide (1-20) and C terminal of contains OmpA_C like domain (78-186). (B) Sequence alignment of Loa22. Loa22 sequence was aligned with other OmpA domain protein including LSS_04621 (Loa22 from *L. santarosai* Shermani), LIC_10191 (Loa22 from *L. interrogan* Copenhageni) ABOmpA (from *A. baumannii*), and ECPAL protein (from *E. coli*). Two pivotal residues (Asp122 and Arg143; hollow square) were highly conserved in Loa22 and other OmpA domain proteins and the two residues were responsible for peptidylglycan (PGN) binding. (C) LAL test of the purified recombinant Loa22. The endotoxin of the recombinant protein was assay by LAL test to measure the endotoxin contamination. ⃰, p<0.05; ⃰ ⃰ ⃰, p<0.001.

**Supplementary Figure 2. AFM images of *L.* *santarosai* and force distribution curves of the Loa22 and LPGN.** (A) The height model of AFM image for *Leptospira*. The *Leptospira* was spray on mica surface and fixed with 1% Glutaraldehyde for 1 h. The fixed *Leptopsira* was incubated with 0.1% TritonX-114 to remove the outer membrane as described in Methods and scanned with AFM in the PBS buffer. (B) 3D view of cell wall structure of *Leptospira*. (C) Force distribution curves of AFM tip interacts to *Leptospira* cell wall, LPGN. (D) Force distribution curves of rLoa22WT modified AFM tip interacts with mica surface. (E) Force distribution curves of rLoa22WT modified AFM tip interacts with *Leptospira* cell wall, LPGN. (F) Force distribution curves of rLoa22D122A modified AFM tip interacts with *Leptospira* cell wall, LPGN. (G) Force distribution curves of rLoa22R143A modified AFM tip interacts with *Leptospira* cell wall, LPGN.

**Supplementary Figure 3. PGN molecules from *Leptosira* mediated co-localization of rLoa22 and TLR2 on HEK293-TLR2 cells.** HEK293-TLR2 cell were cultured to 70% confluence and serum free for 16h before adding the stimulation agents (0.1 μg/ml). The cells were incubated with the stimulation agents for 4h and then fixed to against with the anti-V5 antibody (1:5000) and anti-Loa22 antibody (1:10000). The relative Alex488 and Alex594 conjugated secondary antibodies were used to stained TLR2 and Loa22 proteins, respectively. (A) Purified rLoa22 protein incubated with HEK293-TLR2 cells. (B) LPGN incubated with HEK293-TLR2 cells. (C) rLoa22-LPGN complex incubated with HEK293-TLR2 cells. (D) rLoa22-SPGN incubated with HEK293-TLR2 cells. (E) rLoa22-EPGN incubated with HEK293-TLR2 cells. (F) rLoa22-BPGN incubated with HEK293-TLR2 cells.

**Supplementary Figure S4. Inflammatory responses induced by rLoa22 protein in HEK293-TLR2 cells.** The inflammatory responses from TLR2 signaling such CXCL8/IL8, hCCL2/MCP-1, and hTNF-α were measured after adding the stimulation agents in HEK293-TLR2 cells. (A) Stimulation of the expression of *CXCL8/IL8* mRNA. (B) Stimulation of the expression of CXCL8/IL8 protein. (C) Stimulation of the expression of *hCCL2/MCP-1* mRNA. (D) Stimulation of the expression of hCCL2/MCP-1 protein (E) Stimulation of the expression of *hTNF-α* mRNA. (F) Stimulation of the expression of hTNF-α protein. Purified LipL32 protein was used as positive control and rLoa22WT was treated with heat (100 ^o^C, 30 min) and proteinase K (20 µg/ml at 63 °C for 18 h) to denature and digest the protein. ⃰, p<0.05; ⃰ ⃰, p<0.01.

**Supplementary Figure S5. Inflammatory responses induced by rLoa22 protein in THP-1 cells.** The inflammatory responses from TLR2 signaling such (A) CXCL8/IL8, (B) hCCL2/MCP-1, and (C) hTNF-α were measured after adding the stimulation agents for two hours in THP-1 cells. PBS was used as control and rLipL32 was used as positive control. rLoa22, LPGN, and rLoa22-LPGN were used to stimulate THP-1 to evaluated the protein expression of CXCL8/IL8, hCCL2/MCP-1, and hTNF-α. ⃰, p<0.05.

**Supplementary Figure S6. Transfection efficiencies of TLR1, TLR2, and TLR6 in HEK293 cells.** HEK-293 cells were transfected with TLR2-TLR1, TLR2-TLR6, and TLR2 to measure the transfection efficiencies using flow cytometry. The relative antibodies for TLR1 (#12-4714-81; Thermo Fisher Scientific, Waltham, MA), TLR2 (#11-9922-42; Thermo Fisher Scientific, Waltham, MA), and TLR6 (#MA5-16177; Thermo Fisher Scientific, Waltham, MA) were used to stain the transfected HEK293 cells.

Supplementary Figure 1. Hsu *et al.*, 2020


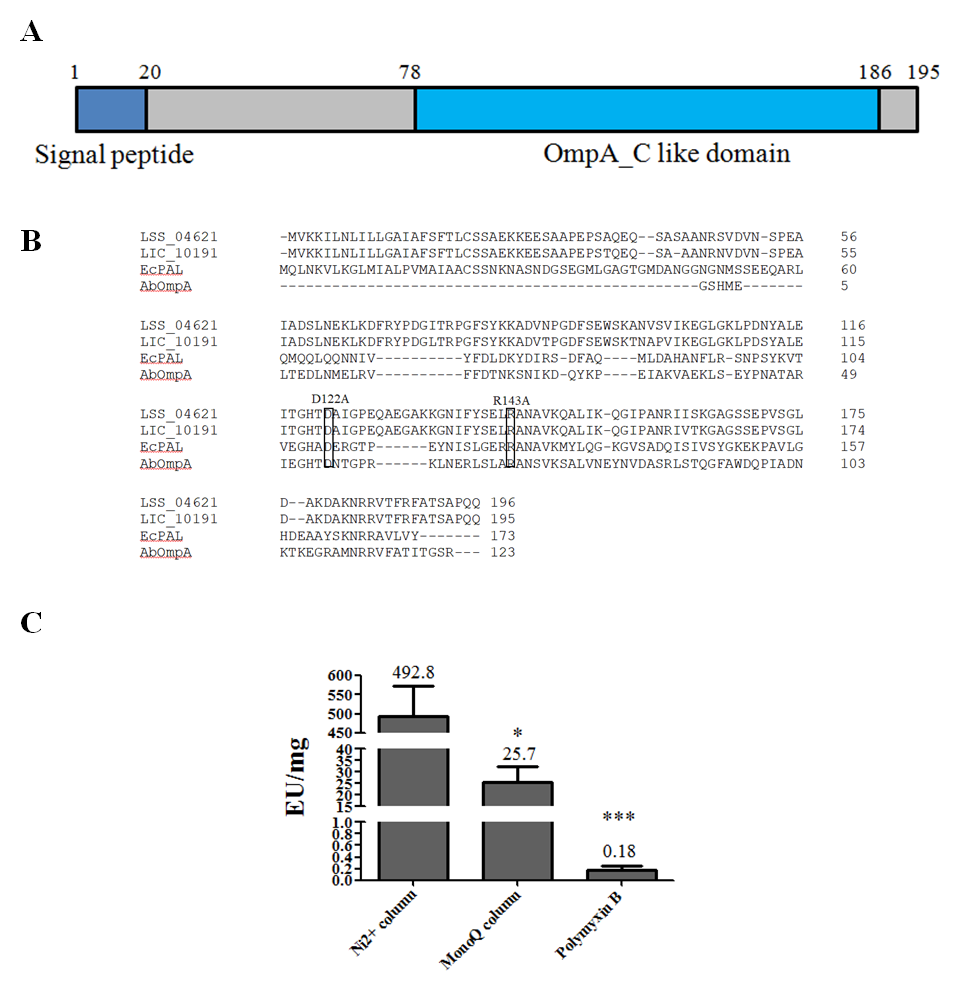


Supplementary Figure 2. Hsu *et al.,* 2020

Supplementary Figure 3. Hsu *et al.,* 2020


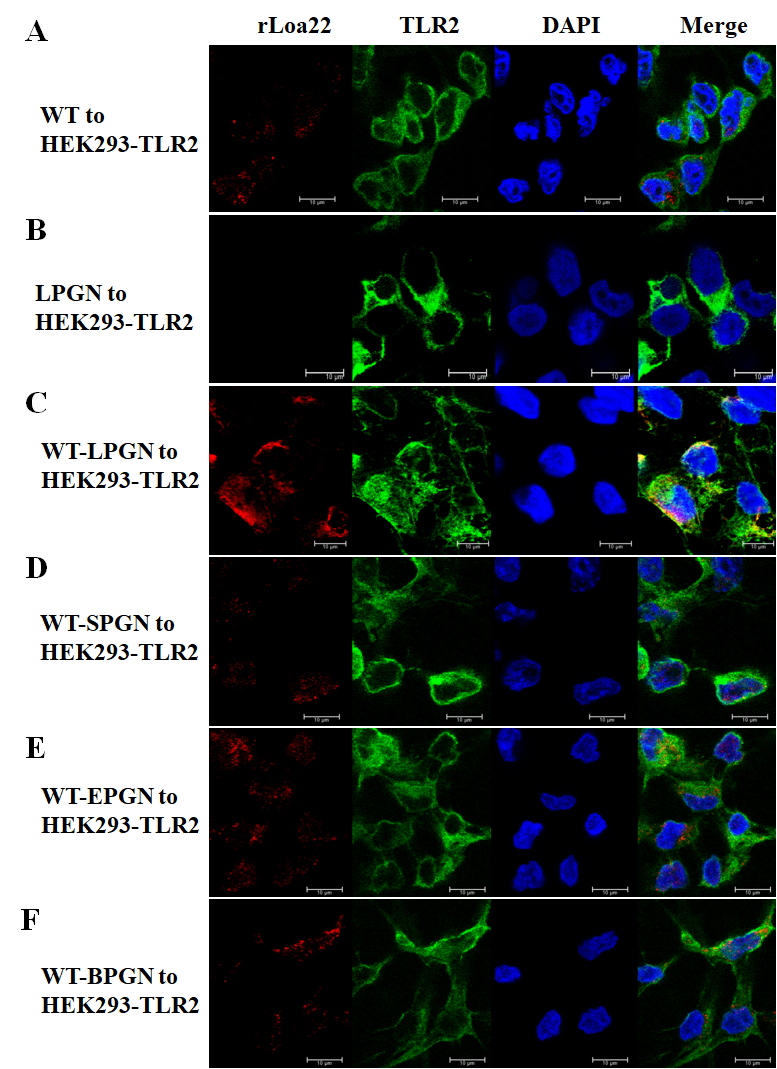


Supplementary Figure 4. Hsu *et al.,* 2020


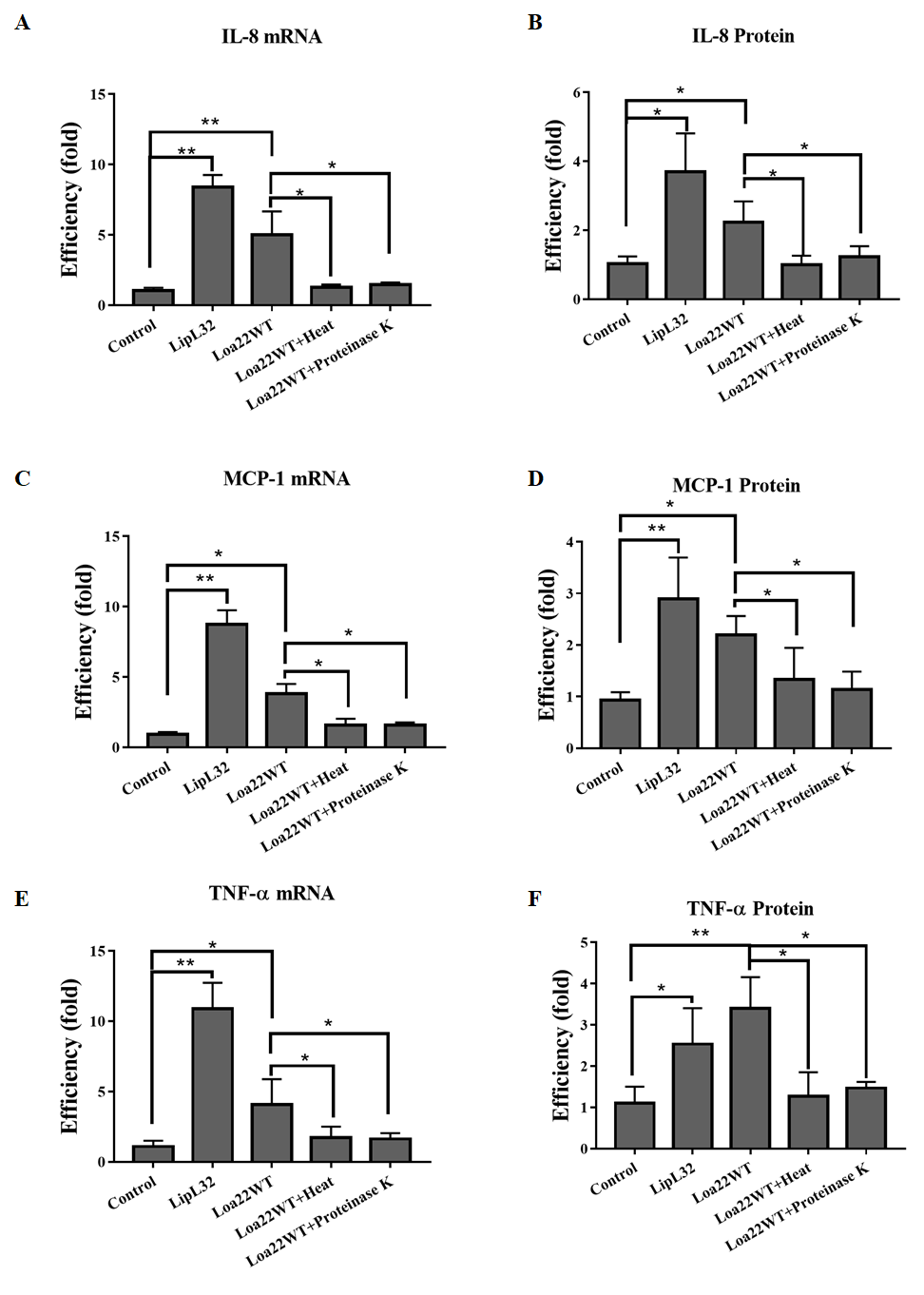


Supplementary Figure 5. Hsu *et al.,* 2020


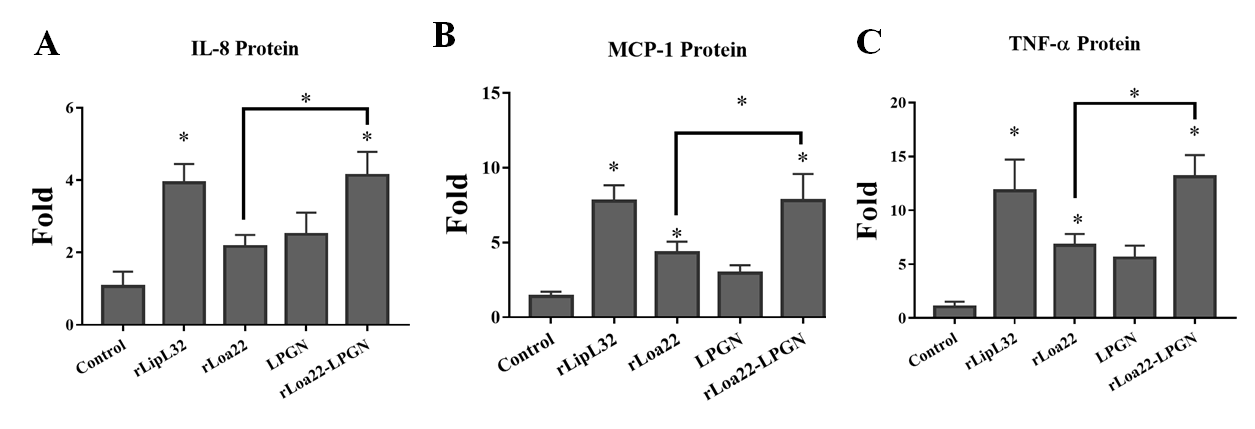


Supplementary Figure 6. Hsu *et al.,* 2020


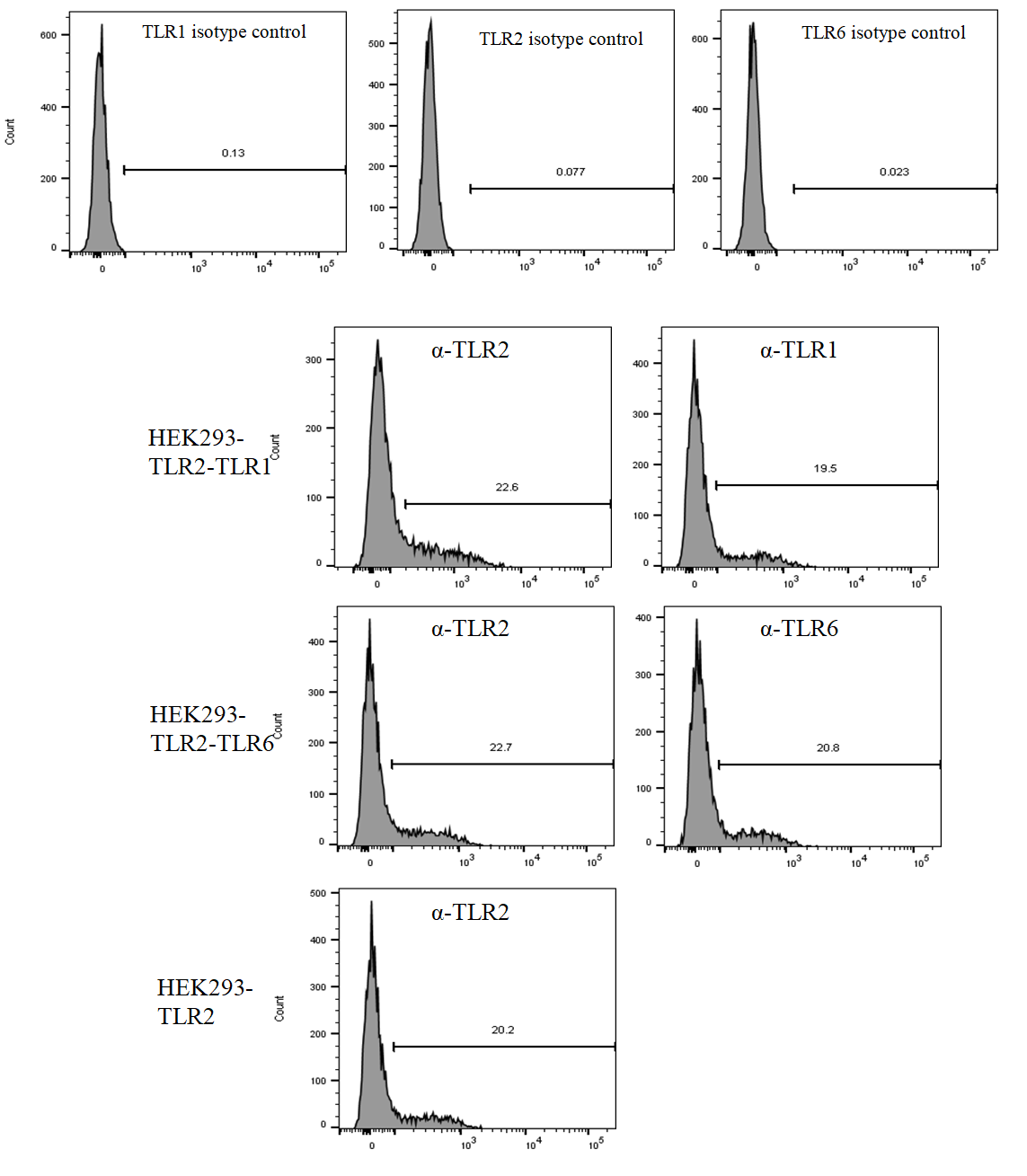


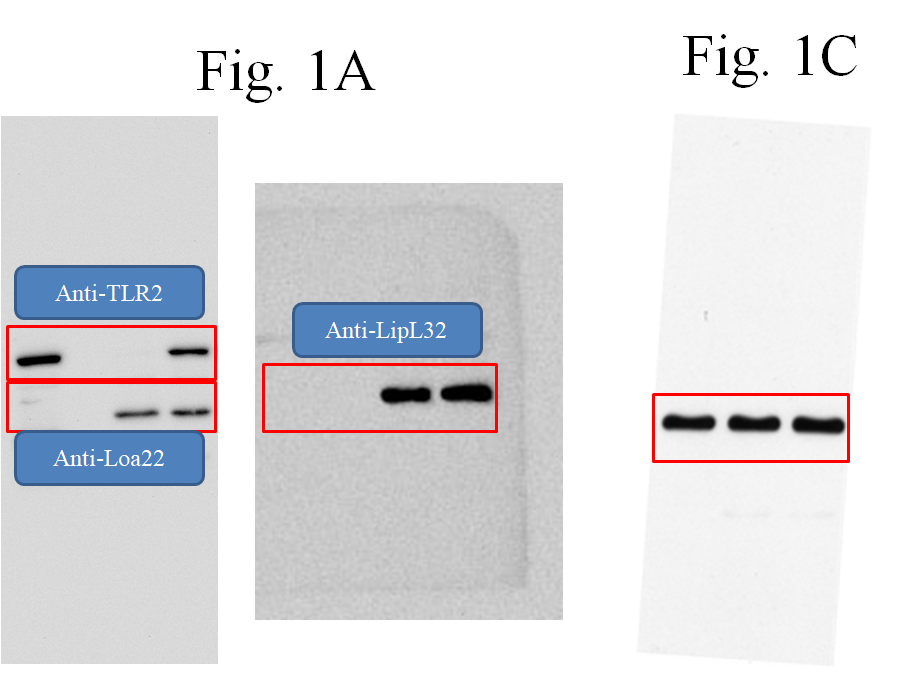


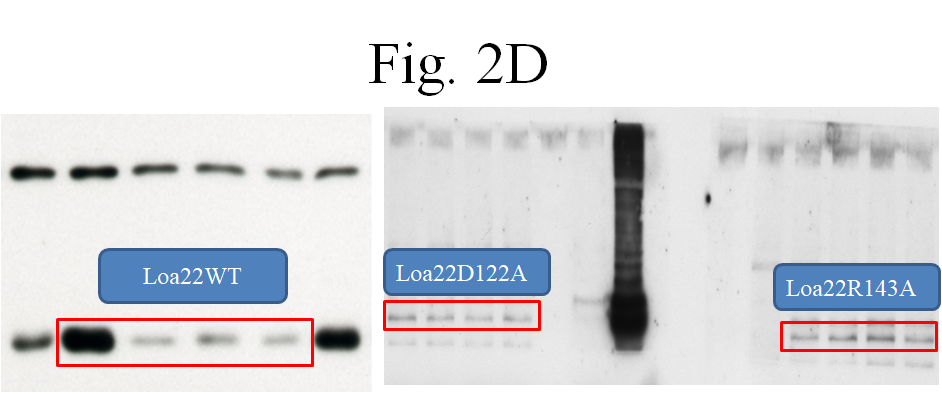


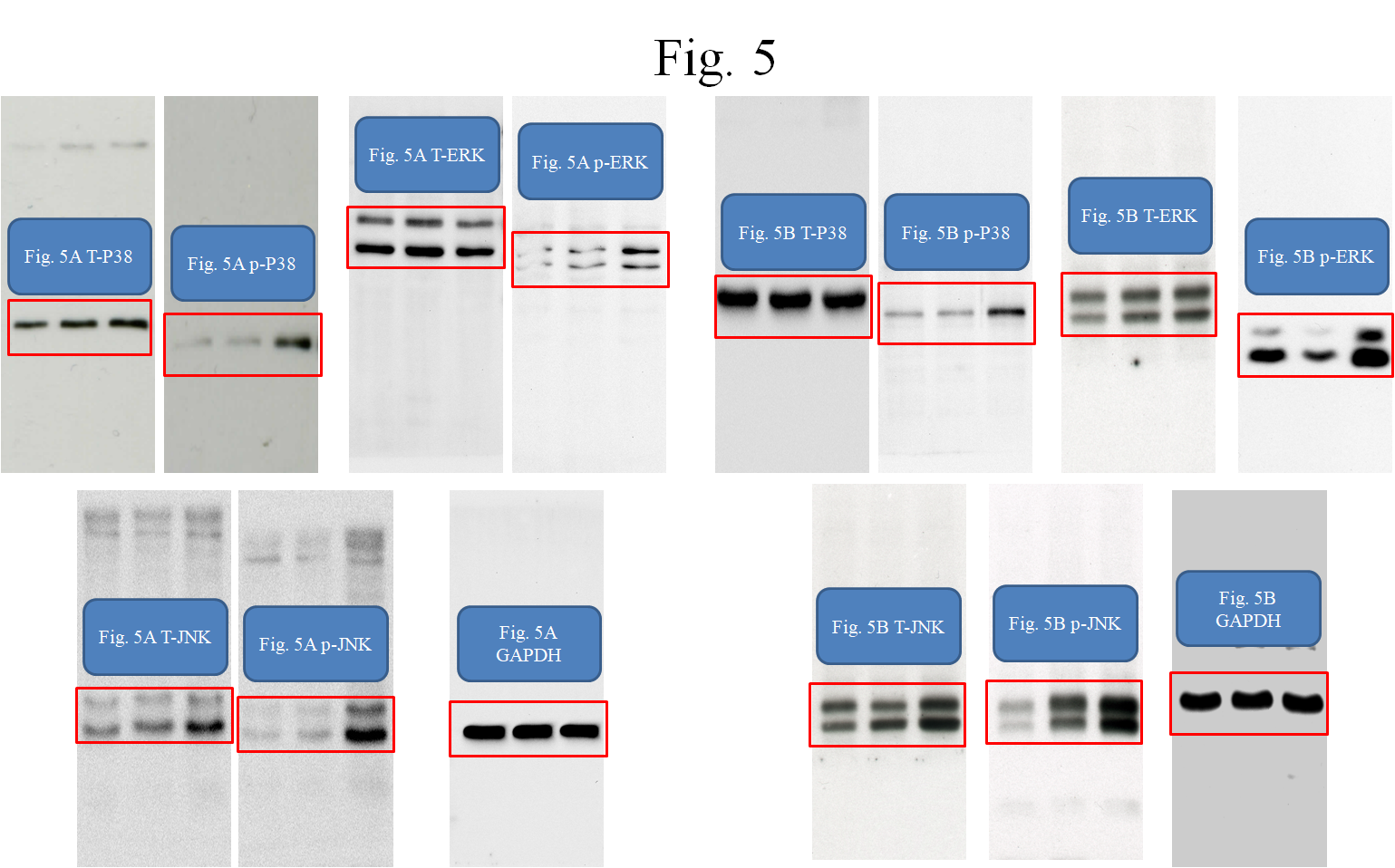

Supplement: Supplementary file 1 — Supplementary Information. [file 41598_2020_79662_MOESM1_ESM.docx]
